# Genome analysis of three *Pneumocystis* species reveals adaptation mechanisms to life exclusively in mammalian hosts

**DOI:** 10.1038/ncomms10740

**Published:** 2016-02-22

**Authors:** Liang Ma, Zehua Chen, Da Wei Huang, Geetha Kutty, Mayumi Ishihara, Honghui Wang, Amr Abouelleil, Lisa Bishop, Emma Davey, Rebecca Deng, Xilong Deng, Lin Fan, Giovanna Fantoni, Michael Fitzgerald, Emile Gogineni, Jonathan M. Goldberg, Grace Handley, Xiaojun Hu, Charles Huber, Xiaoli Jiao, Kristine Jones, Joshua Z. Levin, Yueqin Liu, Pendexter Macdonald, Alexandre Melnikov, Castle Raley, Monica Sassi, Brad T. Sherman, Xiaohong Song, Sean Sykes, Bao Tran, Laura Walsh, Yun Xia, Jun Yang, Sarah Young, Qiandong Zeng, Xin Zheng, Robert Stephens, Chad Nusbaum, Bruce W. Birren, Parastoo Azadi, Richard A. Lempicki, Christina A. Cuomo, Joseph A. Kovacs

**Affiliations:** 1 Critical Care Medicine Department, NIH Clinical Center, National Institutes of Health, Building 10, Room 2C145, 10 Center Drive, Bethesda, Maryland 20892, USA; 2 Genome Sequencing and Analysis Program, Broad Institute of Harvard and Massachusetts Institute of Technology, Cambridge, Massachusetts 02142, USA; 3 Leidos BioMedical Research, Inc., Frederick National Laboratory for Cancer Research, Frederick, Maryland 21701, USA; 4 Complex Carbohydrate Research Center, University of Georgia, Athens, Georgia 30602, USA

## Abstract

*Pneumocystis jirovecii* is a major cause of life-threatening pneumonia in immunosuppressed patients including transplant recipients and those with HIV/AIDS, yet surprisingly little is known about the biology of this fungal pathogen. Here we report near complete genome assemblies for three *Pneumocystis* species that infect humans, rats and mice. *Pneumocystis* genomes are highly compact relative to other fungi, with substantial reductions of ribosomal RNA genes, transporters, transcription factors and many metabolic pathways, but contain expansions of surface proteins, especially a unique and complex surface glycoprotein superfamily, as well as proteases and RNA processing proteins. Unexpectedly, the key fungal cell wall components chitin and outer chain N-mannans are absent, based on genome content and experimental validation. Our findings suggest that *Pneumocystis* has developed unique mechanisms of adaptation to life exclusively in mammalian hosts, including dependence on the lungs for gas and nutrients and highly efficient strategies to escape both host innate and acquired immune defenses.

## Abstract (web summary)

*Pneumocystis jirovecii* is a fungus that can cause life-threatening pneumonia in immunocompromised patients. Here, the authors sequence the genomes of *P. jirovecii* and two other *Pneumocystis* species, and show the unexpected absence of chitin (a near universal fungal cell wall component).

## Graphical abstract

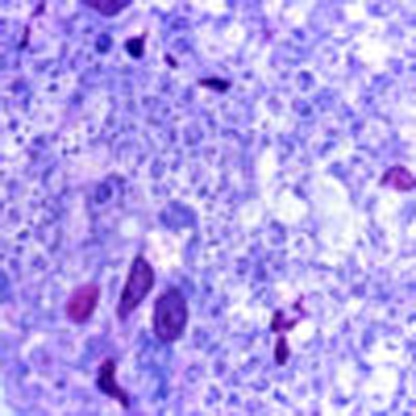

P*neumocystis* organisms were first described in 1909, but *Pneumocystis* became a prominent cause of morbidity and mortality only in the late 20th century, as more humans become susceptible due to HIV/AIDS or immunosuppressive therapies developed for cancer, transplants and inflammatory diseases. The genus *Pneumocystis* comprises a group of highly diversified microorganisms that reside in the lungs of humans and other mammals. *Pneumocystis jirovecii*, the species that infects humans, causes life-threatening pneumonia (termed *Pneumocystis* pneumonia or PCP) exclusively in immunosuppressed patients, and has been responsible for many thousands of deaths over the past 30 years. Despite improvements in diagnosis and treatment, PCP still has an estimated mortality rate of 5–30%. Recently, the frequency of PCP has been increasing in non-HIV immunosuppressed patients, especially renal transplant patients, in whom multiple outbreaks have been reported in the past decade^1^,^2^. Moreover, colonization with *Pneumocystis* is being recognized in an expanding population of patients with underlying pulmonary disease, potentially contributing to accelerated deterioration in pulmonary function^3^. While a number of drugs, including trimethoprim-sulfamethoxazole and atovaquone, are effective in treating and preventing PCP, there have been rising concerns of drug resistance to the best available therapeutic agents^4^.

Unique among fungal species, *Pneumocystis* have adapted to and co-evolved with individual mammalian host species such that each *Pneumocystis* species can infect only a single host species; the basis for this specificity is currently unknown. *Pneumocystis* has two primary stages: spherical cysts, which have a thick cell wall that is rigid, and the more numerous, pleomorphic (amoeboid) trophic forms, which have a thin, flexible cell wall that is often tightly attached to type I pneumocytes in lung alveoli. Animal studies suggest that the cyst form is responsible for transmission between hosts^5^. Although the life cycle remains undefined, it has been hypothesized that trophic forms can undergo binary fission or, alternatively, conjugate and undergo meiosis, leading to the development of cysts with up to eight intracystic bodies, which can subsequently be released as new trophic forms^4^.

Despite its medical importance, our knowledge of the basic biology of *Pneumocystis* remains very limited, in large part due to an inability to reproducibly culture the organism *in vitro*, notwithstanding intense efforts by numerous investigators over the past three decades. Genetic manipulation by transformation is currently untenable. As a consequence, options for developing rational new therapies are currently limited.

To help address these deficiencies, we integrate here whole-genome sequencing and biochemical analysis to gain deeper insights into the biology of *P. jirovecii* (the human pathogen) and two related species infecting mice (*Pneumocystis murina*) and rats *(Pneumocystis carinii*) that serve as important models for studying the pathogenesis and treatment of PCP. While efforts to sequence a *Pneumocystis* genome began two decades ago^6^, the currently available assemblies and annotations of the *P. carinii*^7^ and *P. jirovecii*^8^ genomes remain largely fragmentary (with 4,278 and 358 contigs, respectively) and incomplete; the *P. murina* genome has not been previously sequenced. Furthermore, accurate assembly has been hampered by the presence of highly repetitive sequences in the *Pneumocystis* genome, including the very large multi-copy gene families encoding the major surface glycoproteins (Msg) and kexin proteases, which have been almost entirely excluded from the current genome assemblies of both *P. carinii*^7^ and *P. jirovecii*^8^. Analysis of these draft genomes has provided insights into the sexual reproductive system^9^ and several metabolic pathways such as the amino acid biosynthesis, nitrogen and sulfur assimilation, glyoxylate cycle, myo-inositol biosynthesis, thiamine biosynthesis and purine degradation^8^,^9^,^10^,^11^,^12^,^13^. However, the fragmentary and incomplete status of both genomes has prevented a comprehensive and precise analysis of chromosome structure and gene gain and loss.

In the present study, we generate high-quality genome assemblies that approach the chromosomal level for these three species, and perform RNA-Seq to improve predicted gene structures and to examine gene expression during infection. We also utilize long-read sequencing to characterize the *msg* and kexin gene families, and perform biochemical analysis of the key cell wall components chitin and mannan, to experimentally validate genome predictions. We present the major differences in genome content and functional attributes of *Pneumocystis* in comparison with other fungi and highlight the unique features of *Pneumocystis* species, including cell wall modifications and metabolic shifts that suggest new mechanisms of adaptation to growth as obligate pulmonary pathogens.

## Results

### Conservation and content of highly reduced genomes

We generated three high-quality genome assemblies, including the first *P. murina* genome assembly and new assemblies for *P. carinii* and *P. jirovecii*, all of which are at or near the chromosome level and contain the complete gene set except for a small number of *msg* (in all three species) or kexin genes (in *P. carinii* alone) in several subtelomeric regions that may remain unidentified. Compared with these assemblies, the previously reported assemblies for *P. carinii*^7^ and *P. jirovecii*^8^ are less complete and continuous, with some genes missing entirely or in part, as evidenced by the shorter average gene and protein lengths, the absence of ∼30% of exons in *P. jirovecii* (Supplementary Table 1), and the lack of both *msg* and kexin genes in *P. carinii* and *msg* genes in *P. jirovecii*.

The *P. murina* genome assembly is 7.5 Mb in size across 17 scaffolds, which range in size from 292 to 588 kb (Supplementary Fig. 1a), consistent with the number and size of chromosomes revealed by electrophoretic karyotyping and Southern blotting (Supplementary Figs 2 and 3; Supplementary Note 1). The *P. carinii* genome assembly is 7.66 Mb in size across 17 scaffolds, which range in size from 268 to 635 kb (Supplementary Fig. 1b), in line with previously reported chromosome number and size from electrophoretic karyotyping experiments^14^. The *P. jirovecii* genome assembly is 8.4 Mb in size across 20 scaffolds, ranging in size from 72 to 635 kb (Supplementary Fig. 1c). Previous studies have estimated the *P. jirovecii* genome to be 7.0 Mb in size distributed over 12–13 chromosomes^15^, though this estimate may be inaccurate given the poor quality of available *P. jirovecii* samples.

**Figure 1:**
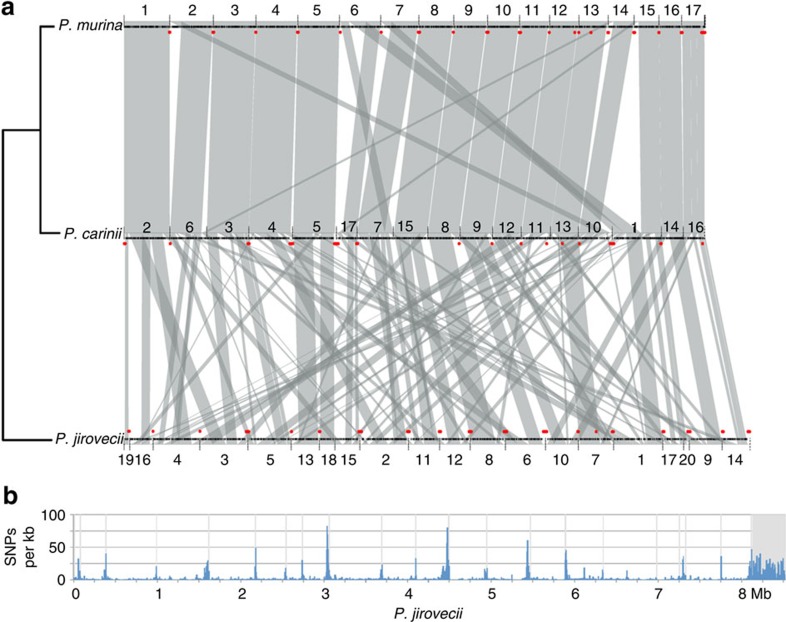
Conservation of *Pneumocystis* genome structure. (**a**) Conserved synteny among three *Pneumocystis* genomes. Shared syntenic regions are depicted with grey boxes. Scaffold numbers are listed on the *x*-axis, and red dots indicate the location of *msg* genes. (**b**) Genome-wide SNP frequency between the *P. jirovecii* isolates from the United States (RU7) and Switzerland (SE8)^8^ with the same scaffold order as in top panel. The region beyond 8 Mb with a high number of SNPs is composed primarily of small scaffolds containing *msg* genes not assembled into the 20 large scaffolds. Using our genome assembly (RU7) as a reference, we identified a total of 24,902 SNPs, or 1 every 337 bases, between these 2 isolates, which are over-represented in subtelomeric regions where *msg* genes are found.

The genomes of *P. murina* and *P. carinii* are very similar in total length, chromosome structure and gene organization, and show limited rearrangements while the genome of *P. jirovecii* is highly rearranged (Fig. 1a; Supplementary Fig. 4; Supplementary Note 2). Between two *P. jirovecii* strains, there is ∼0.3% genetic variation, with subtelomeric regions showing the highest diversity (Fig. 1b) as observed in some other fungi^16^. Despite these rearrangements, conserved syntenic regions include nearly all genes of *P. murina* compared with either *P. carinii* (96.1%) or *P. jirovecii* (92.9%). In contrast, comparison to other phylogenetically related fungi identified only small blocks of three to seven genes with a conserved order in comparison of *P. murina* to *Schizosaccharomyces pombe*^17^ (8.1%) or *Taphrina deformans*^18^ (3.3%). The high number of inter-chromosomal rearrangements in *Pneumocystis* contrasts to the primarily intra-chromosomal pattern reported for *Saccharomycetes*^19^ and other ascomycetes.

Compared with other fungi, all three *Pneumocystis* species have a small genome size and a reduced gene set; between 3,675 and 3,812 genes (including transfer RNA (tRNA) and ribosomal RNA (rRNA) genes) are predicted (Table 1) compared with an average of 5,044 genes in the four related *Schizosaccharomyces* genomes^17^. Other unusual features of the *Pneumocystis* genomes are their possession of only a single copy of rRNA genes (Supplementary Fig. 5), a minimal number of tRNA genes and an extremely low GC content, all of which are among the lowest in eukaryotes (Table 1). These features may reflect a slow transcription and translation machinery (Supplementary Note 3), which, together with the loss of many biosynthetic pathways as discussed below, may lead to the slow growth of *Pneumocystis* organisms as observed in animal models, where doubling times are estimated to be 5–8 days^20^, as well as to the failure to grow *in vitro*. The reduced genome size and content likely reflects adaptation to and dependence on human and other mammalian hosts, as has been previously noted for Microsporidia^21^.

### Gene family expansions and contractions

**Figure 2:**
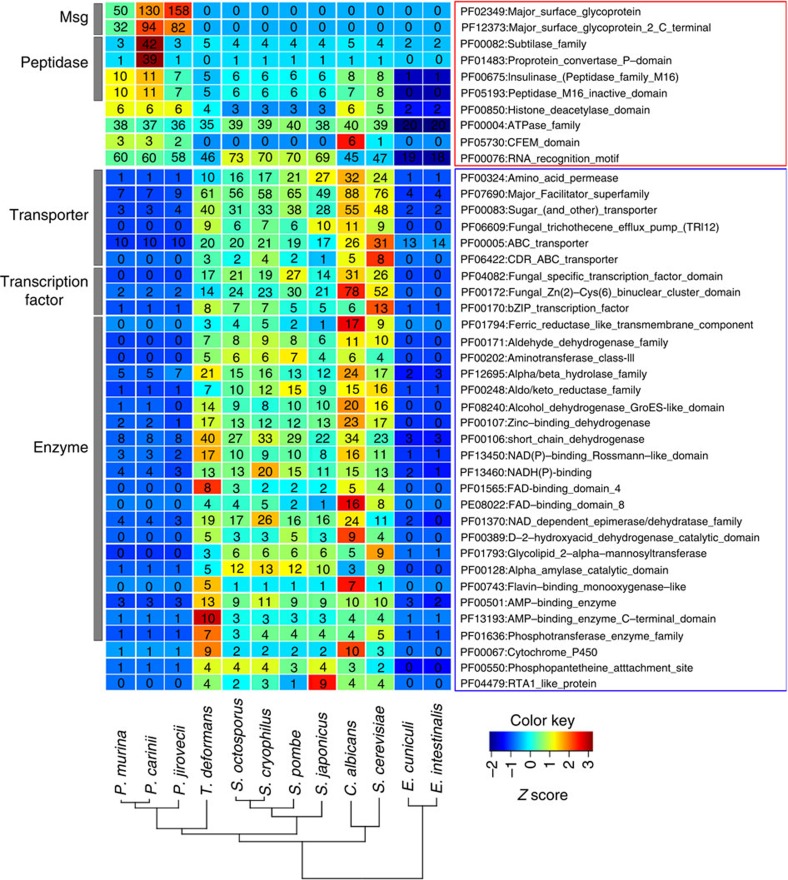
Protein domains depleted or enriched in *Pneumocystis*. Significantly enriched (top panel) or depleted (lower panel) Pfam domains (Fisher's exact test, *q* value<0.05) are included in the heat map if the domains appear at least twice in the following comparisons: *Pneumocystis* versus *Schizosaccharomyces*, *Pneumocystis* versus *Schizosaccharomyces* and *T. deformans*, *Pneumocystis* versus *S. cerevisiae* and *C. albicans*, *Pneumocystis* versus *E. cuniculi* and *E. intestinalis*, *Pneumocystis* versus all others shown. Broader functional categories of proteins are indicated on the left, while specific Pfam domains are listed on the right. The number of proteins containing each domain is indicated within each box for each species. The heat map is colour coded based on a *Z* score, as indicated by the key at the bottom right. Fungal species are ordered based on their phylogenetic relationship as indicated at the bottom.

To examine the predicted functional impact of genome reduction, we examined gene gain and loss in *Pneumocystis* relative to seven closely related Ascomycete species (Supplementary Fig. 6) using protein homology and subcellular localization analysis tools (Supplementary Methods). To identify common features of reduced genomes, we also compared these changes in gene content with those in two microsporidial species^21^, both of which are intracellular organisms and have the smallest known genomes in the fungal kingdom (Fig. 2). Based on a phylogenetic tree inferred from 413 single copy core orthologues (Supplementary Methods), the three *Pneumocystis* species cluster with *Taphrina deformans*^18^ as a sister group to the *Schizosaccharomyces* species (Supplementary Fig. 6). As previously recognized based on mitochondrial genome sequences^22^, *P. murina* and *P. carinii* are more closely related to each other than either is to *P. jirovecii*, although all three species show substantial divergence.

**Figure 3:**
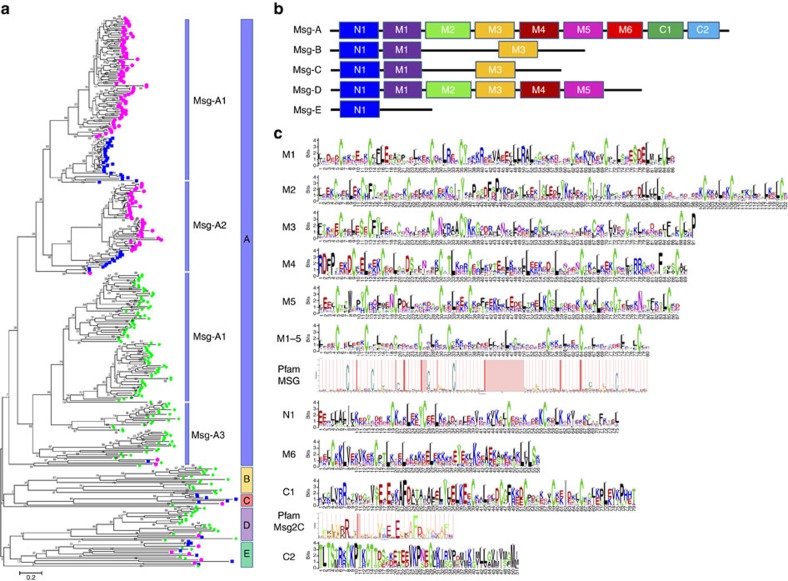
The Msg superfamily in three *Pneumocystis* species. (**a**) Phylogeny of 384 Msg proteins identified in *P. murina* (blue squares), *P. carinii* (pink circles) and *P. jirovecii* (green diamonds). They are classified into five families of Msg-A, -B, -C, -D and -E, as indicated by the vertical bars on the right side. The Msg-A family is further classified into three subfamilies of Msg-A1 (classical Msg genes), Msg-A2 (Msr genes) and Msg-A3 (other Msg-associated genes). (**b**) Schematic representations of conserved domains in five Msg families. (**c**) Sequence logos showing the frequency of amino acid composition in Msg domains. Previously identified Pfam MSG and Pfam Msg2_C domains are included for comparison. Additional information on the Msg domain analysis is provided in Supplementary Note 4.

Protein domains enriched in *Pneumocystis* include host-interacting cell-surface proteins and proteins required for basic cellular functions. The most significantly enriched domains in *Pneumocystis* are the Msg domains, which are shared among *Pneumocystis* species but absent in all other sequenced species. Msg is encoded by a large multi-copy gene superfamily (Fig. 3; Supplementary Table 3; Supplementary Data 1; Supplementary Note 4). Msg genes account for about 3–6% of an otherwise highly reduced genome, suggesting that the encoded proteins play an essential role in the organism's survival. We found extraordinary diversity in genes encoding the Msg superfamily; there were 64 to 179 unique genes per species, which represent the largest surface protein family identified to date in the fungal kingdom^23^ and which show conservation among most Msg families or subfamilies across species, but also species-specific amplifications. Based on domain structure and phylogenetic analysis, the Msg superfamily is classified into five families, designated as Msg-A, -B, -C, -D and -E families (Fig. 3). The high diversity of these Msg families therefore could specify different proteins at the cell surface, both between strains and between species. Msg is thought to play an important role in host–pathogen interactions and potentially facilitates evasion of host immune responses through antigenic variation^23^,^24^. The fact that different *P. jirovecii* strains have unique *msg* repertoires^2^,^25^ dramatically expands the potential for antigenic variation, possibly against T-cell rather than B-cell host responses^26^. Msg antigenic variation by gene recombination is supported by the presence of homologoues to all the key genes involved in homologous recombination in *Saccharomyces cerevisiae* (Supplementary Data 2).

Peptidases of the S8 and M16 families are also highly enriched (Fig. 2; Supplementary Data 3; Supplementary Fig. 7). A large difference between the *Pneumocystis* species is the expansion of the S8B peptidase subfamily (kexin) in *P. carinii*, which contains 39 copies compared with 0–1 copy in *P. murina*, *P. jirovecii* and the other analysed fungi. Kexin is potentially involved in the processing of Msg proteins in the Golgi^27^,^28^,^29^, though in *P. carinii* many of the kexin genes encode proteins predicted to be on the cell surface through a glycosylphosphatidylinositol anchor^29^ (Supplementary Fig. 7b). The protein encoded by the single-copy kexin gene in both *P. murina* and *P. jirovecii* has been predicted to localize to the Golgi apparatus^27^,^28^. Other enriched domains include another cell-surface family, the cysteine-rich CFEM (common in fungal extracellular membrane) domain (Supplementary Fig. 8), as well as proteins involved in regulation of transcription, translation and other cellular activities (histone deacetylase family, ATPase family and the RNA recognition motif (RRM), Supplementary Data 4–6), all of which potentially facilitate the survival of *Pneumocystis* organisms in the host as discussed below.

By contrast, all three *Pneumocystis* species show extensive reduction of multiple gene families, as expected from their small genome size (Fig. 2). The most significantly reduced Pfam domains in *Pneumocystis* comprise the following three major categories: (1) transporters, with <25 transporters in each *Pneumocystis* species compared with 131–217 in other ascomycetes for the 6 depleted Pfam domains; (2) transcription factors, with only 3 genes in each *Pneumocystis* species in the 3 depleted Pfam domains, an order of magnitude fewer than other fungi except for Microsporidia; (3) enzymes, including oxidoreductases, hydrolases, transferases and coenzymes. Most of the significantly depleted domains in *Pneumocystis* are also depleted in Microsporidia (Fig. 2), suggesting common dependencies of these obligate pathogens on their hosts to complement these biologic functions. While additional transcription factor and transporter-associated domains are conserved (Supplementary Data 7 and 8; Supplementary Fig. 9), the total number of transcription factors and transporters in *Pneumocystis* is among the lowest in fungi.

### Substantial reduction and unique features of metabolic pathways

**Figure 4:**
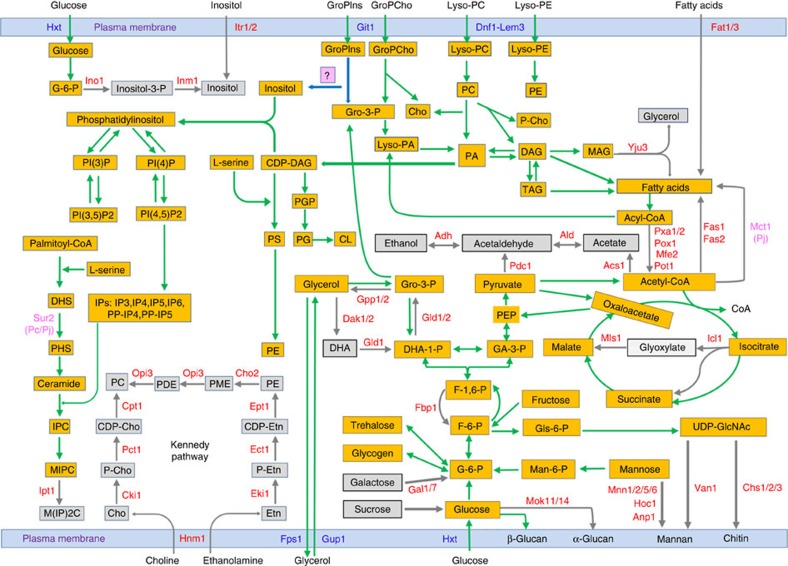
Reduction of carbohydrate and lipid metabolism in *Pneumocystis*. A condensed version of pathways highlights retained (green arrows) and lost (grey arrows) pathways. Enzymes and membrane transporters absent in all three *Pneumocystis* species are highlighted in red font; those retained in all three species are highlighted in blue. Yellow and grey boxes indicate metabolites present and absent, respectively. Some metabolites are included more than once as they interact with multiple pathways, in which case the yellow or grey colouring refers to their role in different pathways. Enzyme Sur2 (in pink) is present in only *P. murina* but not *P. carinii* or *P. jirovecii*. Enzyme Mct1 (in pink) is present in both *P. murina* and *P. carinii* but not *P. jirovecii*. The boxed question mark (‘?') leading to inositol indicates a hypothetical enzyme. The names of enzymes, transporters and metabolites follow the standard abbreviated names for *S. cerevisiae*. The enzyme and transporter names containing two or more digits represent duplicated enzymes and transporters.

**Figure 5:**
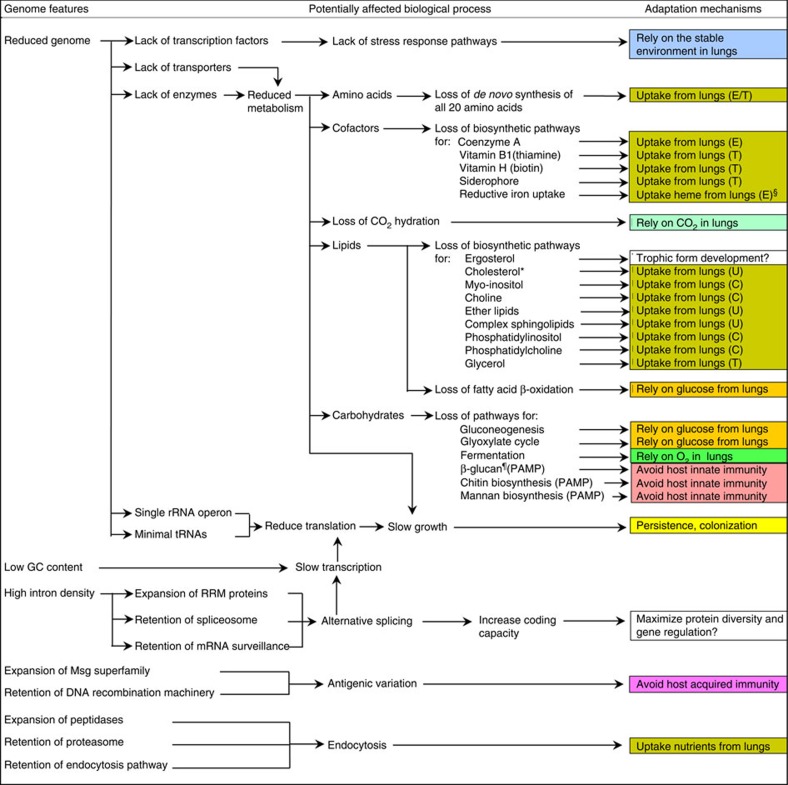
Summary of mechanisms of adaptation to host lungs by *Pneumocystis*. Different mechanisms are highlighted by different colours. Potential mechanisms of uptake of nutrients (which cannot be synthesized *de novo*) include the use of plasma membrane-localized transporters (indicated by T), conversion of other metabolites scavenged from hosts (indicated by C), endocytosis (indicated by E) and unknown (indicated by U). ^§^Potential uptake of haem or haemoglobin from lungs by endocytosis mediated by CFEM domain-containing proteins. *Cholesterol biosynthesis pathway is retained in *P. jirovecii* but lost in *P. murina* and *P. carinii*. ^¶^β-glucan is present in cysts and absent in trophic forms. β-glucan as well as chitin and mannan in other fungal pathogens are known pathogen-associated molecular patterns (PAMP) involved in host immune recognition; none of these components is detected in *Pneumocystis* organisms except for the presence of β-glucan in the cyst form (Figs 6 and 7; Supplementary Fig. 13). *Pneumocystis* is the only fungus identified to date that cannot synthesize chitin.

Since the chromosome-level genome assemblies we generated are more complete than previously reported assemblies^7^,^8^, we mapped all major metabolic pathways for all three *Pneumocystis* species (Figs 4 and 5; Supplementary Figs 10 and 11; Supplementary Data 9–19; Supplementary Note 5). Among the most significantly reduced pathways are those involved in amino acid metabolism. As previously noted^8^,^12^, each *Pneumocystis* species lacks ∼80% of genes involved in *de novo* amino acid synthesis in yeast (Supplementary Data 9). In addition, *Pneumocystis* has impaired capacity for assimilation of inorganic nitrogen and sulfur^10^. Consequently, none of the 20 standard amino acids can be synthesized *de novo* although a few can be synthesized from others. Moreover, in contrast to these earlier reports, we have identified only 1 potential amino acid transporter (Ptr2), which is predicted to localize to the plasma membrane in each species compared with over 20 such transporters in yeasts. Nevertheless, intracellular transport appears highly conserved; nearly half of the 26 mitochondrion- and vacuole-associated amino acid transporters in yeast are preserved. While it is believed that polyamines are ubiquitous in all organisms and serve diverse functions, none of the three *Pneumocystis* genomes encodes any of the enzymes necessary for *de novo* synthesis of polyamines, though there is one potential polyamine transporter in each species, consistent with previous *in vitro* studies of *P. carinii*^30^. All three *Pneumocystis* species have retained the genes required for *de novo* nucleotide synthesis but are missing nearly all the genes for nucleotide salvage pathways (Supplementary Data 10).

For carbohydrate metabolism, loss of a subset of pathways further highlights nutritional dependency. All three *Pneumocystis* species have a full complement of genes necessary for uptake and catabolism of glucose via glycolysis and the tricarboxylic acid (TCA) cycle (Fig. 4; Supplementary Data 11). In addition, all three species have all the enzymes necessary to convert fructose and mannose to glucose, and to synthesize and utilize glycogen and trehalose. However, key enzymes that convert galactose and sucrose to glucose are missing. Additional notable losses include two enzymes for glyoxylation^8^, one key enzyme for gluconeogenesis, and all enzymes for pyruvate fermentation. These findings, together with the identification of almost all genes involved in oxidative phosphorylation (Supplementary Data 16), suggest that energy production in *Pneumocystis* largely relies on glucose through oxidative pathways.

Lipid metabolism genes in *Pneumocystis* are also greatly reduced in number, thus resulting in distinctive differences in predicted lipid content compared with other fungi (Fig. 4; Supplementary Data 12). All three *Pneumocystis* species are able to synthesize fecosterol and episterol, but unable to convert them to ergosterol, which potentially accounts for the resistance of *Pneumocystis* to classical antifungal agents as noted previously^11^. Moreover, only *P. jirovecii* but not *P. murina* or *P. carinii* can synthesize cholesterol^13^ (Supplementary Fig. 11). These findings support the hypothesis that *P. murina* and *P. carinii*, but not necessarily *P. jirovecii*, may scavenge cholesterol from their hosts^31^, though the genes involved in sterol uptake have not been identified. Utilization of cholesterol rather than ergosterol may be contributing to a less rigid cell wall, thus allowing development of the trophic form of *Pneumocystis* since cholesterol-containing membranes are more flexible than ergosterol-containing membranes. All three *Pneumocystis* species lack not only the *de novo* synthesis pathways for myo-inositol, choline, complex sphingolipids, ether lipids, phosphatidylinositol, phosphatidylcholine and fatty acids (the cytosolic pathway involving *fas1* and *fas2* genes) but also the transporters for direct uptake of these lipids from external sources (Fig. 4; Supplementary Data 12). Nevertheless, alternative mechanisms could supply cells with phosphatidylinositol, phosphatidylcholine, inositol and choline (Fig. 4; Supplementary Note 5).

Strikingly, most of the genes involved in fatty acid β-oxidation are missing in all three species, suggesting that fatty acids are not an energy source for *Pneumocystis*, further supporting a high reliance on glucose as the main energy source. Of note, all three *Pneumocystis* species lack the enzymes for synthesis of glycerol from glycerone-phosphate or monoacylglycerol but encode homologues of yeast proteins (Gup1 and Fps1) responsible for glycerol uptake and export (Fig. 4; Supplementary Fig. 9b). Although glycerol is the only non-sugar carbon source that can enter into the TCA cycle, and is also required for synthesis of glycerolphospholipids, these two transporters may also play an important role in maintaining osmotic balance given the loss of critical cell wall components as discussed below.

Cofactor metabolism is also largely reduced in *Pneumocystis* (Supplementary Data 13). All three *Pneumocystis* species are missing almost all enzymes required for *de novo* synthesis and membrane transport of pantothenate, but retain all enzymes needed to convert pantothenate to CoA, as well as a mitochondrial carrier protein (Leu5) for CoA (Supplementary Fig. 10), implying possible scavenging of pantothenate or its downstream metabolites from the host by other mechanisms (for example, endocytosis. Fig. 5; Supplementary Data 14). All three *Pneumocystis* species lack enzymes required for *de novo* synthesis of vitamins B1 and H, as well as ubiquinone and siderophores, but have a potential plasma membrane transporter for each of these cofactors (Supplementary Data 13; Fig. 4; Supplementary Fig. 9c,d). *P. jirovecii* also lacks enzymes for *de novo* synthesis of NAD, while retaining one salvage pathway using exogenous nicotinic acid mononucleotide imported by a transporter (Tna1). In contrast, both *P. murina* and *P. carinii* have all the enzymes and the transporter needed for *de novo* synthesis and salvage of NAD. All three *Pneumocystis* species show a near complete absence of proteins necessary for reductive iron assimilation and siderophore biosynthesis^32^ (Supplementary Data 13). Since each *Pneumocystis* species encodes five proteins with cysteine-rich CFEM domains (Supplementary Fig. 8), it is possible that, like *Candida albicans*^33^, *Pneumocystis* is able to scavenge iron from host haem and haemoglobin using one or more of these proteins as an extracellular haem receptor.

Our analysis suggests that endocytosis may serve as a mechanism for *Pneumocystis* to obtain nutrients in the absence of multiple different receptors. In support of this hypothesis, we found that each *Pneumocystis* species encodes nearly all proteins involved in clathrin-dependent endocytosis (Supplementary Data 14). In addition, genes encoding various degradative enzymes (including proteases, lipases, ATPase families and proteasome shown in Supplementary Data 3,5,12 and 15) and transporters localized in mitochondria and vacuoles are expanded or retained, and some of them are highly expressed (Supplementary Data 20), while biosynthetic pathways for many nutrients (including amino acids, lipids and cofactors, as noted above) and plasma-membrane-associated transporter families are completely lost or reduced. Loss of these transporters may reflect low concentrations of their targets in the host lung milieu.

### Lack of chitin, outer chain N-mannans and α-glucan in cell wall

Fungal cell walls typically are composed primarily of chitin, chitosan, glucans, mannans and glycoproteins, which are covalently cross-linked together to protect the cell from changes in environmental stresses, while allowing the organism to interact with its environment. The structure and biosynthesis of such cell walls are unique to fungi and thus serve as an excellent target for antifungal agents. The formation and assembly of the *Pneumocystis* cell wall remain poorly understood, though numerous studies have found it to be rich in glycoproteins and, in cysts only, β-glucans^34^,^35^.

**Figure 6:**
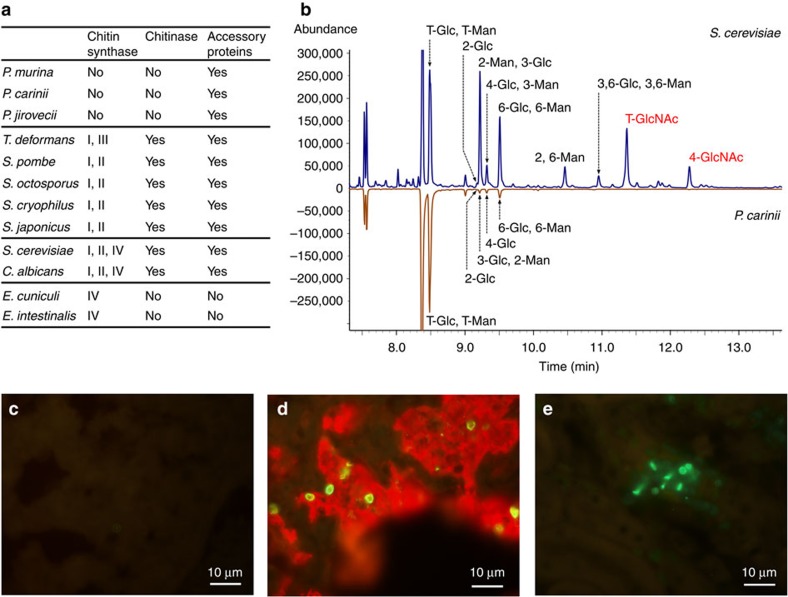
Analysis of chitin in *Pneumocystis* and related fungi. (**a**) Enzymes and accessory proteins involved in chitin metabolism in fungi. *Pneumocystis* genomes do not encode any chitin synthase or chitinase, which are present in other fungi, but retain genes encoding four accessory proteins (Supplementary Data 17). (**b**) Gas chromatograms of partially methylated alditol acetates of *P. carinii* and *S. cerevisiae* (control) cell walls. Terminal and 4-linked N-acetylglucosamine signals (T-GlcNAc and 4-GlcNAc in red font) were detected in *S. cerevisiae* but not in *P. carinii*. Glucose (Glc) and mannose (Man) signals were detected in both species. (**c**–**e**) Detection of chitin with recombinant chitin-binding domain (Alexafluor 488) using *P. murina*-infected lung tissue (**c**) and *C. albicans*-infected kidneys (**e**) as a positive control. *Pneumocystis* organisms are demonstrated in **d** by dual staining with anti-Msg (red), which labels both trophic forms and cysts, and a dectin-Fc construct (green), which labels β-1,3-glucan in cysts. Chitin staining is absent in *P. murina* but readily detected in *C. albicans*, while β-1,3-glucan is easily seen in *P. murina*. Original magnification, × 400; scale bar, 10 μm.

Remarkably, none of the three *Pneumocystis* species encodes the key enzyme chitin synthase required for chitin synthesis, or chitinases involved in chitin degradation during cell wall remodelling (Fig. 6; Supplementary Data 17). The absence of these two gene families strongly suggests that *Pneumocystis* does not contain chitin in its cell wall. While each *Pneumocystis* genome does encode homologues of a few accessory proteins not directly involved in chitin synthesis or degradation (such as Chs5 reported elsewhere^36^), none of these shares any signature protein domains found in chitin synthase or chitinase (Supplementary Fig. 12). In *Saccharomyces cerevisiae*, Chs5 functions as a component of the exomer complex involved in export of chitin synthase and other membrane proteins^37^; in *Pneumocystis* Chs5 could serve only the latter function, given the absence of chitin synthase. While the detection of chitin in *P. carinii* has been previously reported^36^,^38^ based on reactivity with non-specific lectins such as wheat germ agglutinin, this staining may be detecting other molecules based on the absence of chitin synthases in the genome.

We assayed for the presence of chitin in the *Pneumocystis* cell wall using a recombinant chitin-binding domain (CBD). While we found strong reactivity with *Candida* cell walls by *in situ* labelling, reactivity with *Pneumocystis* was totally absent (Fig. 6). In addition, we directly examined the cell wall content of partially purified *Pneumocystis* organisms and cultured *S. cerevisiae* cells using mass spectrometric analysis. No chitin-related oligosaccharides were identified in *Pneumocystis*, while they were detected in *S. cerevisiae*; glucan-related oligosaccharides were detected in both (Supplementary Note 6). The combination of these genomic and experimental results demonstrates that *Pneumocystis* is the first identified member of the fungal kingdom that does not have chitin.

β-glucans have been identified in the wall of the cyst form of *Pneumocystis*, but are absent from the more abundant trophic form^34^,^39^. *Pneumocystis* genomes encode all the enzymes required for β-1,3- and β-1,6-glucan synthesis and degradation^34^,^39^,^40^,^41^ (Supplementary Data 18). However, *Pneumocystis* species do not have any genes involved in synthesis and degradation of α-glucan, which has been found in many fungi, and which can block innate immune recognition by the β-glucan receptor^42^.

**Figure 7:**
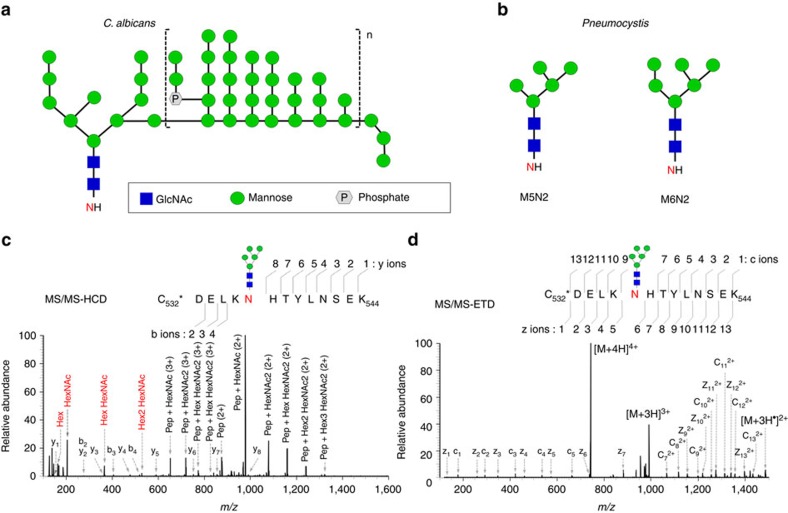
Lack of hyper-mannose (mannan) glycosylation in *Pneumocystis*. (**a**) Diagram of N-linked mannan structure in *C. albicans*, based on ref. 43. (**b**) Diagram of N-linked glycans in *Pneumocystis*, which lack the α-1,6-linked mannose backbone as well as α-1, 2- and α-1,3- linked mannose outer chains seen in *C. albicans* (square brackets). (**c**,**d**) Representative results of tandem mass spectrometry (MS/MS)-higher energy collisional dissociation (HCD) and electron transfer dissociation (ETD) analysis of an N-linked glycopeptide carrying Hexose5 HexNAc2 (M5N2) from one Msg isoform (T552_03736) in *P. carinii*. (**c**) MS/MS-HCD spectrum of glycopeptides showing the detection of glycan oxonium ions in the low mass region at *m/z* 163.0603, 204.0868, 366.1398 and 528.1929 (indicated in red font). A series of fragment ions dues to neutral loss of the glycan moiety were observed as the main fragment ions in the HCD spectrum. Trace amounts of y-type and b-type peptide fragment ions were detected, confirming the sequence of the peptide backbone. (**d**) MS/MS-ETD spectra of peptide fragment ions with minimal neutral loss of glycan moiety. All expected peptide c-type and z-type fragment ions were detected except c8 fragment ion, confirming the peptide sequence with high confidence, as well as the site and mass of the glycosylation modification.

Although all *Pneumocystis* species have abundant surface glycoproteins, especially Msg, little is known about their glycosylation state and other post-translational modifications. We found that *Pneumocystis* species encode enzymes required for synthesis of the N- and O-linked glycan core structure (containing up to nine mannose residues), which are localized to the endoplasmic reticulum, but lack genes for enzymes residing in the Golgi apparatus, which add mannose outer chains, including all the enzymes comprising mannan polymerase complex I and complex II, and α-1,6-, α-1,2- and α-1,3-mannosyltransferase (Supplementary Data 19). The absence of these enzyme genes suggests that, unlike other fungi, *Pneumocystis* cell wall proteins including Msg are not highly mannosylated. N-linked profiling of PNGase-F-released N-glycans showed that M5N2 is the predominant N-linked glycan on *P. carinii* Msg proteins (Supplementary Fig. 13; Supplementary Note 7). Although trace amounts of M6N2 to M9N2 were detected as minor components, mannan type N-glycans with more than nine mannose residues were not detected. Moreover, glycopeptide mapping of Msg tryptic digest by using liquid chromatography-tandem mass spectrometry (LC-MS/MS) identified 31 N-linked glycans in 15 Msg isoforms, all of which carried M5N2 as the predominant component and only 1 of which carried M6N2 as an additional, minor component (Fig. 7; Supplementary Fig. 13; Supplementary Data 21). The lack of N-linked outer chain mannan may allow the organism to avoid recognition by innate immune responses, since in *Candida* such mannosylation is required for recognition by dectin-2, DC-SIGN and the macrophage mannose receptor of dendritic cells, while mutants that can synthesize only the core structure are poorly recognized^43^,^44^.

### Transcription enrichment during infection

The expression level of each annotated gene was estimated using RNA-Seq data from three heavily infected animals each for *P. murina* and *P. carinii*. Overall, we find evidence of expression for nearly all genes (99%, see Methods) but variation in expression level over 5 orders of magnitude. Using Gene Set Enrichment Analysis (GSEA^45^), we identified functional categories that were enriched in highly expressed genes in each species (Supplementary Data 20; Supplementary Fig. 14). The most highly enriched categories include Msgs and predicted secreted proteins (24–25 other than Msgs in each species). The enrichment of the latter suggests that additional secreted proteins may play an important role during infection. In addition, many functions involved in general metabolism of RNA and proteins, including the RNA RRM and LSM domain, are enriched among highly expressed genes in *Pneumocystis*.

Each *Pneumocystis* genome contains an exceptionally high intron density, with an average of 5 introns per gene, similar to only a few fungal genomes such as *Cryptococcus* with high levels of splicing and only slightly fewer than that in mammalian genomes (7–9 introns per gene)^46^. This is unusual for highly compacted fungal genomes where intron loss is usually observed, as in *S. cerevisiae*^47^ and Microsporidia^48^. The transcription and splicing process for genes with many introns requires higher energy and cellular resources; this appears correlated to the relative expansion and high-level expression of RRM domain-containing genes (Fig. 2; Supplementary Fig. 14; Supplementary Data 6) and genes involved in spliceosome and mRNA surveillance in all *Pneumocystis* genomes (Supplementary Data 22). Using the RNA-Seq data, we identified high-level alternative splicing events in *P. murina* and *P. carinii*, with intron retention being the most common (detected for 42–49% of introns) and other types being infrequent (≤3% of introns for each type) (Supplementary Data 23). We assembled full-length alternatively spliced isoforms without premature termination codons for 263 and 275 genes of *P. murina* and *P. carinii*, respectively, though there is no functional enrichment of these genes. The high rate of intron retention correlates with the presence of all the components of the nonsense-mediated mRNA decay machinery in each *Pneumocystis* species (Supplementary Data 22). Alternative splicing of intron-containing genes could increase transcript diversity and regulate gene transcription or mRNA stability in this otherwise reduced genome, as suggested in earlier studies of *P. carinii*^49^ and other organisms^50^.

### Adaption to a host lung environment

Our genome analysis has revealed new insights into the dependence of *Pneumocystis* on mammalian hosts. *Pneumocystis* has been identified almost exclusively in the lungs of humans and other mammals, where it remains extracellular but preferentially attaches to type I pneumocytes. Although an environmental reservoir has been hypothesized, there is no convincing evidence of such a reservoir; the current genome data strongly suggest that the entire life cycle occurs in the host. Genes involved in mating and meiosis are present and transcribed in all three *Pneumocystis* species (Supplementary Data 24), suggesting that sexual reproduction is actively occurring in lung tissue, as postulated in previous studies^9^,^11^. Thus, *Pneumocystis* presumably must obtain nutrients and proliferate in the lung environment, and at the same time it must withstand host defenses for sufficient periods to allow direct transmission to another susceptible host.

During the process of adaption as an obligate pathogen, the *Pneumocystis* genome has contracted substantially compared with other fungi, though not to the extent of Microsporidia, which are intracellular organisms, while *Pneumocystis* are exclusively extracellular. The retention of all components for glucose uptake and catabolism while the glyoxylate and gluconeogenesis pathways were lost strongly suggests a high dependency on glucose from the host as the primary energy and carbon sources. Transcriptional regulation is also greatly simplified, implying that the host lung provides an optimal environment for *Pneumocystis*, with little need for sensing and responding to stress, as evidenced by the loss of genes involved in the pH sensing, osmotic and oxidative stress responses and cAMP-mediated signalling (Supplementary Data 25). The loss of the pyruvate fermentation pathway and other genes known to be essential for anaerobic growth (Supplementary Data 11 and 26) likely reflects adaptation exclusively to aerobic respiration in the lung environment with a stable oxygen supply. Another very unusual loss in *Pneumocystis* is the gene encoding carbonic anhydrase (Supplementary Data 9), which catalyses the interconversion between CO_2_ and bicarbonate, a key mechanism for providing bicarbonate to cells and for regulation of intracellular pH, and which is widely distributed in all life kingdoms^51^. This enzyme may be unnecessary in the CO_2_-rich lung environment (5–6%), while its absence suggests *Pneumocystis* is unable to survive under ambient air conditions (0.03–0.04% CO_2_).

## Discussion

Our study suggests that *Pneumocystis* is highly adapted to existence in the host lung, with strict dependence on the mammalian host for nutrients and a stable environment, while utilizing efficient strategies for immune evasion, thus facilitating colonization and persistence in its host. Among the most striking findings in this study is the loss of two important fungal cell wall components, chitin and outer chain N-mannans, both predicted by genome analysis and further supported by biochemical assays. Furthermore, β-1,3-glucan has previously been shown to be absent from the cell wall of the trophic form of *Pneumocystis*^34^,^39^, which is ∼10- to 20-fold more abundant than the cyst form. While β-glucan is present and exposed in some *Pneumocystis* cysts, we have found that in most cysts the β-1,3-glucan is masked by Msg and other surface proteins (unpublished observations). Given that chitin, β-glucans and mannan are all known pathogen-associated molecular patterns (PAMPs) that trigger innate host defense responses through interactions with host pattern recognition receptors (PRRs) such as dectin-1 and DC-SIGN^43^,^52^, their simultaneous absence or masking may be beneficial in evading or delaying recognition of *Pneumocystis*, especially the trophic form, by the innate immune system. Antigenic variation provided by Msg isoforms extends potential immune evasion to include adaptive immune responses. Retention of β-glucan in cysts presumably is critical to organism survival, possibly by providing a rigid spherical cell wall that is aerodynamically efficient for transmitting infection to other hosts, while protecting the organism from the harsher environmental conditions outside the lung^5^. The absence of chitin and β-glucan on the cell wall (Fig. 6) suggests that trophic forms are more fragile than other fungi. Greater malleability of the trophic cell wall could facilitate intimate contact with host cell membranes, and improve the ability of *Pneumocystis* to obtain nutrients by direct cell-to-cell contact or by endocytosis. The evolutionary adaptation by *Pneumocystis* is in sharp contrast to other pathogenic fungal species that normally live outside the mammalian host while being able to infect different host species and different tissues.

The availability of three high-quality *Pneumocystis* genomes provides important information about the biology of these species, and potentially provides insights that will guide developing *in vitro* culturing of this organism, a critical goal of the *Pneumocystis* research community. Moreover, given the loss of many biosynthetic and metabolic pathways, the remaining pathways, including the limited number of transporters (Supplementary Table 4), provide attractive targets for anti-*Pneumocystis* drug development.

## Methods

### *Pneumocystis* organisms from rodents and humans

*P. murina*-infected lung samples were obtained from CD40 ligand knock-out female mice^26^. *P. carinii*-infected lung samples were obtained from immunosuppressed Sprague-Dawley male rats^24^. *P. jirovecii*-infected autopsy lung samples were from one patient with AIDS, who was infected with only one *P. jirovecii* strain based on genotyping at five different genomic loci^53^. Animal and human subject experimentation guidelines of the National Institutes of Health were followed in the conduct of these studies.

### Preparation of genomic DNA samples

*Pneumocystis*-infected lung tissues were cut into small pieces, homogenized in Qiagen Tissuelyser (5 times for 20 s at 1/30 frequency), then centrifuged at 15,000*g* for 6 min. The pellet was resuspended in Trypsin/EDTA Solution (Lonza), incubated at 37 °C for 30 min and centrifuged at 15,000*g* for 6 min. The pellet was washed once in PBS, resuspended in lysis buffer containing 2.9% (w/v) collagenase type I (Gibco) and 0.1% (w/v) DNase I (Sigma), incubated at 37 °C for 30 min, and centrifuged at 15,000*g* for 6 min. This sequential enzyme digestion was expected to remove most of the host cells and DNA as well as potentially some of *Pneumocystis* trophic forms. The pellet was washed three times in PBS, resuspended in 100 μl of 0.5% (w/v) Zymolyase solution (Zymo Research) and incubated at 37 °C for 1 h. Genomic DNA was extracted using the MasterPure Yeast DNA Purification Kit (Epicentre). RNA was removed using DNase-free RNAase (Epicentre).

All DNA samples were analysed by quantitative real-time PCR (qPCR) assays using fluorescence resonance energy transfer probes^54^ to measure the fraction of *Pneumocystis* DNA compared with host DNA in each sample. In qPCR for both *P. murina*^20^ and *P. carinii*^54^, the target was the single-copy dihydrofolate reductase (*dhfr*) gene of *Pneumocystis* and the target for the host was a highly conserved region of the single-copy polycystic kidney disease 1 (*pkd1*) gene^22^. The targets for *P. jirovecii* and human qPCR were the *msg* gene family^55^ and the single-copy β-globin gene^56^, respectively. The purity of each DNA sample was estimated by the ratio of *Pneumocystis* to host genome copy number and based on an estimated genome size of 8 Mb for *Pneumocystis* and 2.7–3.3 Gb for the host. The qPCR results were validated by 454 or Illumina MiSeq sequencing of selected samples. DNA samples extracted from enriched *P. murina* or *P. carinii* preparations contained up to 90% *Pneumocystis* DNA, and from enriched *P. jirovecii* preparations, 10–25% *P. jirovecii* DNA, while those extracted directly from heavily infected lung tissues contained <0.4–1% *Pneumocystis* DNA.

### *P. murina* genome sequencing and assembly

To assess the quality of the *P. murina* DNA preparations for whole-genome sequencing, small-scale sequencing was first conducted using a 454 GS FLX Titanium Sequencer (Roche Applied Science) at Leidos, Inc. (Frederick, MD) according to the 454 standard shotgun sequencing protocols. A total of 4 million reads (with a mean length of 344 bases) were generated, with ∼40% of them having blast hits with *P. carinii*^7^. Subsequently, deep sequencing was performed using Illumina HiSeq technology at the Broad Institute (Cambridge, MA). Seven different *P. murina* DNA preparations (each with 60–90% purity for *P. murina* by qPCR) were used to construct small insert libraries; each was sequenced, and the library (preparation B123 from a single mouse, center project G11228) with the lowest percent of contaminating host mouse DNA was used for assembly. From this library, a total of 34 million 101 base paired-end reads with a mean insert size of 153 bases were generated. A second larger insert library (preparation C2 from another mouse, center project G11230) with a mean insert size of 1,247 bases was prepared, and a total of 83 million 101 base paired-end reads were generated. After removal of mouse sequences (version mm9) and *P. murina* mitochondrial sequences^22^, these reads were assembled with Allpaths (version R37380) with default parameters^57^; the resulting assembly was screened for contaminating sequences (mouse and bacteria) and to remove *P. murina* mitochondrial sequences^22^ by aligning to the non-redundant nucleotide database from NCBI and by removing the scaffolds with high coverage that matched non-fungal organisms. The draft assembly of 24 scaffolds was further improved using long 454 reads and PCR to support scaffold joins. The final assembly contained 17 scaffolds. All internal contig gaps were closed by PCR and Sanger sequencing. The ends of scaffolds without *msg* genes or telomere repeats were extended by PCR and/or PacBio sequencing (Supplementary Methods).

### *P. carinii* genome sequencing and assembly

After preliminary 454 sequencing demonstrated a purity of 63%, 1 *P. carinii* DNA preparation (no. B80 from 2 heavily infected rats, 80% purity for *P. carinii* by qPCR) was used to construct 2 libraries with a mean insert size of 180 bases and 793 bases, respectively. Each was sequenced using 101 base paired-end reads on the Illumina MiSeq platform at the Broad Institute, and, after removal of host rat sequences and *P. carinii* mitochondrial sequences^22^, assembled using AllPaths-LG (version R47825) with default parameters, resulting in a total of 53 scaffolds. After joining scaffolds using long 454 reads and confirming by PCR, the final genome assembly contained 17 scaffolds. All internal contig gaps were closed by PCR and Sanger sequencing. The ends of six scaffolds overlapped with the seven telomere sequences reported elsewhere^58^; they were merged and confirmed by PCR and/or aligning merged scaffolds with Illumina and 454 raw reads. The ends of several scaffolds without *msg* genes, kexin genes or telomere repeats were extended by PCR and/or PacBio sequencing (Supplementary Methods).

### *P. jirovecii* genome sequencing and assembly

Three enriched DNA samples (RU7, RU12 and RU817) from a single autopsy lung sample RU (1.4–2 μg each, with 10–25% *P. jirovecii* DNA by qPCR) were used to construct three libraries (156 base average insert size); each was sequenced on the Illumina HiSeq platform at the Broad Institute. To improve on initial assemblies with high numbers of contigs, additional sequence was generated from hybrid-selected DNA samples as previously described^59^; 120 base oligo bait probes were designed to target regions present in the genome assembly as well as to pull in uncovered regions and transcripts; probes were designed for: existing assemblies^8^ including higher probe density at contig ends, unassembled reads^8^ that did not match the human genome, assembled RNA-Seq transcripts^8^ that did not match existing assemblies or the human genome and unanchored *msg* sequences^25^. Host sequences were removed by aligning to human genome assembly 19 (GRCh37/hg19), and *P. jirovecii* mitochondrial sequences^22^ were removed. The remaining 101 base paired-end reads were assembled separately with Spades 2.5.1 (at the Broad Institute), Spades 3.0 (at Leidos) and Abyss (Biowulf at NIH), which resulted in 400, 149 and 312 contigs, respectively. All these contigs were merged with Sequencher (Gene Codes Co., Ann Arbour, MI), resulting in a total of 20 scaffolds. All internal gaps were closed by PCR and Sanger sequencing. Scaffolds were validated by examining raw reads aligned by Seqman Pro (DNAStar, Madison, WI) to ensure there were no false joins. To improve the coverage of *msg* genes in the assembly, PacBio sequencing was performed as described in Supplementary Methods.

### Chromosome electrophoresis and Southern hybridization

To compare the *P. murina* assembly with chromosome number and length, we used contour clamped homogeneous electrical field (CHEF) electrophoresis to separate chromosomes. *P. murina* organisms were partially purified from fresh mouse lungs by Ficoll-Hypaque density gradient centrifugation^60^, and then processed for CHEF^61^ using the CHEF Yeast Genomic DNA Plug Kit (Bio-Rad). Briefly, partially purified *P. murina* organisms were embedded in 0.8% CleanCut agarose, treated with 24 U ml^−1^ proteinase K overnight at 50 °C, then washed four times in 1 × Wash Buffer and stored at 4 °C. Electrophoresis was performed in 1% agarose gels (14 × 21 cm) using the CHEF DR II apparatus (Bio-Rad). Gels were run in 0.5 × TBE buffer for 144 h at 135 V and 12.5 °C, with a 50 s initial pulse that was gradually increased to 100 s. The gel was stained with ethidium bromide (Sigma), then DNA was transferred to Nytran membranes (Schleicher & Schuell) under neutral conditions^61^. We used two blots prepared from the same DNA plug, and each blot was hybridized consecutively to different probes, with stripping of the blot between hybridizations. All probes were PCR-amplified double-stranded DNA fragments labelled using the PCR DIG Probe Synthesis Kit (Roche Applied Science) except for the probe for telomere repeats, which was a synthesized oligonucleotide labelled using the DIG Oligonucleotide Tailing kit (Roche Applied Science). All primer and probe sequences are provided in Supplementary Data 27. Hybridization and signal detection were performed by using the DIG Probe Hybridization system (Roche Applied Science) as described previously^22^.

### RNA-Seq, expression levels and gene prediction

Three independent samples of *P. murina* and *P. carinii* organisms were partially purified from heavily infected lungs of three mice and rats, respectively, by Ficoll-Hypaque density gradient centrifugation^60^. The partially purified pellets were expected to contain both cyst and trophic forms. Total RNA was isolated using the RNeasy Mini Kit (Qiagen). The ratio of *Pneumocystis* to host RNA was estimated to be from 1:4 to 8:1 based on the density of RNA bands in agarose gels for the host 28S rRNA and *Pneumocystis* 26S rRNA. Strand-specific libraries were constructed using the dUTP second strand marking method^62^,^63^, except as noted below. For all samples, poly(A) RNA was purified using the Dynabeads mRNA Purification kit (Life Technologies), with two rounds of selection (with bead regeneration), resulting in <5% rRNA as measured by the Bioanalyzer RNA 6000 Pico chip (Agilent) program. The resulting mRNA (200 ng per sample) was treated with the Turbo DNA-free kit (Ambion), checked by qPCR for the absence of detectable genomic DNA (Supplementary Table 5), and fragmented in 1 × RNA fragmentation buffer (Affymetrix) for 4 min at 80 °C. After first-strand complementary DNA (cDNA) synthesis a 2.0 × volume of RNAClean SPRI beads (Beckman Coulter Genomics) clean-up was used instead of phenol/chloroform/isoamyl alcohol (25:24:1) extraction and ethanol precipitation. Size selection after adapter ligation was done with two 0.7 × Ampure beads (Beckman Coulter Genomics) clean-up steps and eight cycles of PCR were used to generate the library. Libraries were sequenced on the HiSeq2000 platform generating an average of 26.1 million paired 76 base reads for each of the 6 samples.

To examine expression levels, RNA-Seq reads from each of the three samples for each organism were aligned to the extracted coding DNA sequences (CDSs) using bowtie^65^. The alignment bam files were then used to quantify transcript abundances by RSEM^66^. We selected the top 5% of expression levels to examine functional enrichment by GSEA^45^. For each organism, we examined GSEA of gene sets classified based on Pfam, TIGRFAM, KEGG and SignalP functional annotations. We also ran GSEA on some manually curated gene sets such as *msg* genes.

The relative expression level for each gene was expressed as fragments per kb of exon per million fragments mapped (FPKM). For both *P. murina* and *P. carinii*, 99% of all annotated genes were expressed (FPKM>2), with a median coverage of 151 FPKM in both species. Further, expressed genes showed good alignment depth across the transcript, with 98% of all genes in each species having at least fivefold RNA-Seq depth. All genes without detectable expression (26 in *P. murina* and 23 in *P. carinii*) contain short (average size of ∼250 bp, potentially pseudogenes) or incomplete CDSs.

The *P. murina* and *P. carinii* genomes were annotated using a combination of expression data (RNA-Seq), homology information and *ab initio* gene finding methods (Supplementary Methods) as previously described^67^. These methods were also used to annotate the RU assembly of *P. jirovecii*, with the exception that RNA-Seq data was not used (Supplementary Methods). Gene sets were compared based on functional annotation and used as the basis for syntenic and phylogenetic analysis (Supplementary Methods).

### Analysis of strain variation in *P. jirovecii*

To examine genome-wide single nucleotide polymorphisms (SNPs) between sequenced isolates, the RU7 assembly generated in this study was used as the reference compared with the SE8 raw reads^8^. SE8 reads were retrieved from NCBI (ERR135854 to ERR135863) and converted to fastq format. Individual fastq files were concatenated, and the full read set aligned to the RU7 assembly using BWA-MEM^68^ and converted to sorted bam using SAMtools^64^. This resulted in very high alignment depth across all assembly bases, with a median alignment depth of 1,046.

The Genome Analysis Toolkit^69^ (GATK) v2.7–4-g6f46d11 was used to call both variant and reference bases from the alignments. Briefly, the Picard tools (http://picard.sourceforge.net) AddOrReplaceReadGroups, MarkDuplicates, CreateSequenceDictionary and ReorderSamwere were used to preprocess the alignments, followed by GATK RealignerTargetCreator and IndelRealigner for resolving misaligned reads close to indels. Next, GATK UnifiedGenotyper (with the haploid genotype likelihood model (GLM)) was run with both SNP and INDEL GLM. We additionally ran BaseRecalibrator and PrintReads for base quality score recalibration on sites called using GLM SNP and re-called variants with UnifiedGenotyper emitting all sites. A final filtering step was used to remove any position that was called by both GLMs (that is, incompatible indels and SNPs) and to remove positions with low read depth support (<10). The 24,902 SNPs were then mapped to the gene set predicted on the SE8 assembly; a total of 14,135 SNPs are located in CDS regions, with substitutions affecting protein coding regions as follows: 7,410 nonsynonymous, 6,690 synonymous, 26 nonsense and 9 extensions. SNP density across the SE8 assembly was calculated for 5-kb windows.

### Detection of chitin by fluorescence labelling

The cDNA sequence encoding the CBD of *Bacillus circulans* chitinase A1 gene^70^ (2,183–2,338 bp, GenBank accession code M57601.1) was optimized for bacterial expression (GenScript USA Inc.) and modified by the addition of ATG and 3 HA tags. The modified sequence was synthesized (GenScript USA Inc., Supplementary Fig. 15) and cloned into pET-28 vector (EMD Biosciences). The cDNA construct was transformed into *Escherichia coli* strain BL21 (DE3) RIL (Agilent technologies) and expressed as a His tag fusion protein. The expressed protein was purified in two steps, first using anti-CBD antibody (New England Biolabs) that was immobilized using AminoLink Plus Immobilization Kit (Thermo Scientific), and then the Hispur cobalt purification kit (Thermo Scientific). The purified protein was biotinylated using Lightning-Link Biotin conjugation kit (Type A) (Innova Biosciences Ltd) and used for fluorescence labelling (Histoserv, Inc., Germantown, MD) of *P. murina*-infected lung tissue sections fixed in Histochoice (Amresco, Inc.). Alexafluor 488-conjugated streptavidin was used for the detection of CBD. *Pneumocystis* organisms were detected by an anti-Msg antibody^26^ and cysts by a dectin-Fc recombinant protein (kindly provided by Dr Chad Steele, the University of Alabama at Birmingham, Alabama)^71^. Mouse kidney sections infected with *Candida* (kindly provided by Dr Michail Lionakis, the National Institute of Allergy and Infectious Diseases, Bethesda, Maryland)^72^ were used as a positive control for chitin staining (Fig. 5).

### Chitin glycosyl linkage analysis

To prepare cell wall fraction*, P. carinii* organisms were partially purified from infected rat lungs by Ficoll-Hypaque density gradient centrifugation^60^; *S. cerevisiae* organisms were obtained from Stratagene (strain YPH499) and grown in standard yeast culture. Cells were resuspended in denaturing buffer (2% SDS, 1 mM EDTA in 50 mM Tris pH 8.0) and heated at 100 °C for 10 min. The cell wall pellet was recovered by centrifugation at 5,000*g* for 5 min., washed twice with PBS, and dried in a speed vacuum concentrator.

For chitin analysis, cell wall pellets were incubated with 0.1% (w/v) chitinase (Sigma) in 50 mM sodium acetate buffer, pH 5.6, at 37 °C for 72 h. Insoluble materials after the initial digestions were further treated sequentially with pronase (Roche), lyticase (Sigma) and chitinase to look for residual chitin. Protein digestion was performed using 0.01% (w/v) pronase in Tris-HCl buffer pH 8.2 with 10 mM CaCl_2_ at 37 °C for 48 h. Glucan digestion was performed with 0.04% (w/v) lyticase in 50 mM sodium phosphate buffer, pH 7.5 at 37 °C for 24 h. Chitinase digestion was performed as above. After each digestion, the enzyme was inactivated by heating at 100 °C for 5 min.

Digested cell wall components were analysed by matrix-assisted laser-desorption ionization time-of-flight MS (MALDI/TOF-MS) and nanospray ionization MS (NSI-MSn). An aliquot of supernatant after each enzyme digestion was analysed by MS to determine oligosaccharide content. Prior to MS analysis, enzymes were removed by passing through a C18 Sep-PAK cartridge. The flow-through was collected in 5% acetic acid and lyophilized. The dried samples were permethylated using previously published methods^73^ and profiled by MS. MALDI/TOF-MS was performed in the reflector positive ion mode using DHBA (α-dihyroxybenzoic acid, 20 mg ml^−1^ solution in 50% methanol/water) as a matrix. The spectrum was obtained by using an AB SCIEX TOF/TOF 5800 (AB SCIEX). NSI-MSn analysis was performed on an LTQ Orbitrap XL mass spectrometer (Thermo Fisher) equipped with a nanospray ion source. Permethylated sample was dissolved in 1 mM NaOH in 50% methanol and infused directly into the instrument at a constant flow rate of 0.5 μl min^−1^. A full Fourier transform MS spectrum was collected at 30,000 resolution. The capillary temperature was set at 210 °C and MS analysis was performed in the positive ion mode. For total ion mapping (automated MS/MS analysis), an *m/z* range of 200–2,000 was scanned with ion trap MS mode in successive 2.8 mass unit windows that overlapped the preceding window by 2 MU.

For determination of glycosyl linkages, partially methylated alditol acetates (PMAA) were prepared from fully permethylated glycans. The permethylated glycans were hydrolyzed with HCl/water/acetic acid (0.5:1.5:8, by vol.) at 80 °C for 18 h, followed by reduction with 1% NaBD_4_ in 30 mM NaOH overnight, then acetylation with acetic anhydride/pyridine (1:1, v/v) at 100 °C for 15 min and analysis by gas chromatograph-MS (GC–MS) on an Agilent 7890A GC interfaced to a 5975C MSD (mass selective detector, electron impact ionization mode). The separation of the PMAA was performed on a 30-m SP2331 fused silica capillary column (Supelco) for the neutral sugar derivatives, and a DB-1 (Agilent) column for amino sugar derivatives.

### Msg protein glycosylation identification

To purify *P. carinii* Msg proteins, partially purified *P. carinii* organisms^60^ were resuspended in 2% SDS in 50 mM Tris-HCl buffer, pH 8.0, boiled for 10 min and centrifuged. The supernatant was collected. The extraction procedure was repeated two more times, the supernatants were pooled and SDS was removed using the SDS-Out SDS Precipitation Kit (Thermo Scientific). Solubilized Msg was affinity purified using an anti-Msg monoclonal antibody (RA-E7, gift of Drs Peter Walzer and Michael Linke^74^, the University of Cincinnati, Cincinnati, Ohio) immobilized on to AminoLink plus column (AminoLink plus immobilization kit) following manufacturer's instructions. The protein extract was diluted with an equal volume of Tris buffered saline and incubated with the immobilized antibody in the column by end-over-end mixing overnight at 4 °C. The column was then drained and washed with 0.1 M sodium phosphate buffer pH 6.9. The Msg was eluted with 150 mM ammonium hydroxide and fractions were neutralized with 1 M NaH_2_PO_4_. The fractions containing Msg were pooled and concentrated using Microcon-30 kDa centrifugal filter unit (Millipore) and the buffer was exchanged to 0.1 M sodium phosphate buffer pH 6.9. Aliquots were stored at −80 °C.

Purified Msg proteins were subjected to glycopeptide detection by LC-MS. Glycoproteomic analysis was carried out as previously described^75^. Briefly, the tryptic digested peptides of purified Msg proteins were separated with a Magic C18 column (15 cm × 75 μm, Bruker-Michrom, CA) and analysed with an Orbitrap Fusion (Thermo Scientific) mass spectrometer after Msg was denatured, reduced, alkylated and desalted. Gradient elution was performed with a 30 min linear gradient of 5–35% acetonitrile in 0.1% formic acid at a flow rate of 300 nl min^−1^. The MS data were processed automatically by a data-dependent MS scan pipeline, which integrated three dissociation methods, including higher energy collisional dissociation (HCD), electron transfer dissociation (ETD) and collision-induced dissociation (CID). In this pipeline, the full MS spectrum was first acquired from most abundant ions between *m/z* 350 and *m/z* 1,550 with a 3-second cycle time at 120,000 resolutions in FT mode. Subsequently the acquired MS data were assessed by MS/MS with HCD-product-dependent ETD or HCD-product-dependent ETD/CID mode with most abundant ions within 3-s cycle time. For the HCD-product ion trigger MS/MS, glycan oxonium ions at *m/z* 204.0867 (HexNAc), *m/z* 138.0545 (HexNAc fragment) or *m/z* 366.1396 (Hexose-HexNAc) in the HCD spectra were used to trigger ETD or CID acquisition. The MS/MS were measured at a resolution of 15,000.

For the mapping of glycopeptides from *P. carinii* Msg digests, LC-MS data were analysed using Byonic software (Protein Metrics) and the data annotation by the software was manually validated. Byonic parameters were set to allow 3.0 p.p.m. of precursor ion mass tolerance and 3.0 p.p.m. of fragment ion tolerance with monoisotopic mass. Digested peptides were allowed with up to three missed internal cleavage sites, and the differential modifications of 57.02146 and 15.9949 Da were allowed for alkylated cysteine and oxidation of methionine, respectively. Protein database used for protein blast included the protein set of the rat genome at NCBI (version 104) and of the *P. carinii* genome B80 generated in this study. Glycan database used was 309 mammalian N-glycan database (Default database in the Byonic software). Released N-linked glycan analysis of Msg was performed prior to the glycopeptide mapping.

Data annotation by the software was further filtered as follows. Any protein identification with FDR >1%, log probability <4 or best Byonic score <500 was excluded. Moreover, any glycopeptide spectra annotations with Byonic score <400 were excluded. Glycopeptide identifications that remained after these strict filters were manually validated by looking at fragment ions matched to the theoretical glycopeptide fragmentation, the presence of a series of expected glycan oxonium ions and neutral loss of the glycan moiety for MS/MS-HCD data.

## Additional information

**Accession codes:** Illumina raw reads have been deposited in the NCBI Sequence Read Archive for the genome sequencing of *P. murina* (accession codes SRX248091 and SRX1435036), *P. carinii* (accession codes SRX387635 and SRX387636) and *P. jirovecii* (accession code SRX387638). Illumina raw reads for RNA-Seq of *P. murina* and *P. carinii* have been deposited in the NCBI Sequence Read Archive under accession code PRJNA292577. Genome assemblies and annotations have been deposited in the NCBI BioProject database with accession codes PRJNA70803 (*P. murina*), PRJNA223511 (*P. carinii*) and PRJNA223510 (*P. jirovecii*). All sequence data has been linked to NCBI Umbrella project PRJNA223519.

**How to cite this article:** Ma, L. *et al*. Genome analysis of three *Pneumocystis* species reveals adaptation mechanisms to life exclusively in mammalian hosts. *Nat. Commun.* 7:10740 doi: 10.1038/ncomms10740 (2016).

## Supplementary Material

Supplementary Information Supplementary Figures 1-15, Supplementary Tables 1-5, Supplementary Notes 1-7, Supplementary Methods and Supplementary References

Supplementary Data 1 Members of the msg superfamily in three *Pneumocystis* species.

Supplementary Data 2 *Pneumocystis* genes involved in DNA recombination.

Supplementary Data 3 *Pneumocystis* peptidase family.

Supplementary Data 4 *Pneumocystis* genes encoding histone deacetylase.

Supplementary Data 5 *Pneumocystis* genes encoding ATPase.

Supplementary Data 6 *Pneumocystis* RRM-domain-containing proteins.

Supplementary Data 7 Transcription factors in *Pneumocystis* and other fungi.

Supplementary Data 8 *Pneumocystis* transporters.

Supplementary Data 9 *Pneumocystis* genes involved in amino acid metabolism.

Supplementary Data 10 *Pneumocystis* genes involved in nucleotide metabolism.

Supplementary Data 11 *Pneumocystis* genes involved in carbohydrate metabolism.

Supplementary Data 12 *Pneumocystis* genes involved in lipid metabolism.

Supplementary Data 13 *Pneumocystis* genes involved in cofactor metabolism.

Supplementary Data 14 *Pneumocystis* genes involved in clathrin-dependent endocytosis.

Supplementary Data 15 *Pneumocystis* genes involved in proteasome.

Supplementary Data 16 *Pneumocystis* genes involved in oxidative phophorylation.

Supplementary Data 17 Fungal genes involved in chitin metabolism.

Supplementary Data 18 *Pneumocystis* genes involved in glucan metabolism.

Supplementary Data 19 *Pneumocystis* genes involved in glycan metabolism.

Supplementary Data 20 GSEA of categories enriched in genes highly expressed during *Pneumocystis* infection.

Supplementary Data 21 Glycopeptide mapping of *P. carinii* Msg protein digests.

Supplementary Data 22 *Pneumocystis* genes involved in spliceosome.

Supplementary Data 23 *Pneumocystis* genes with alternative splicing.

Supplementary Data 24 *Pneumocystis* genes involved in mating and meiosis.

Supplementary Data 25 *Pneumocystis* genes involved in cell signaling and stress responses.

Supplementary Data 26 *Pneumocystis* genes involved in anaerobic growth.

Supplementary Data 27 Oligonucleotide primer and probe sequences used in PCR and DNA hybridization.

## Figures and Tables

**Table 1:** Comparison of *Pneumocystis* and related fungal genomes.

Species*	**Chromosomes or scaffolds (count)**	**Genome size (Mb)**	**GC content (%)**	**Protein coding genes (count)**	**Exons per gene (count)**	**tRNA genes (count)**	**rRNA genes**† **(count)**	**Intergenic distance (mean, bp)**
*P. murina*	17	7.50	26.9	3623	6.08	47	5	423
*P. carinii*	17	7.66	33.2	3646	5.97	45	5	430
*P. jirovecii*	20	8.40	28.4	3761	5.78	46	5	483
*T. deformans*	394	13.36	49.5	4661‡	2.10	169	5	827
*S. pombe*	3	12.57	36.1	5155	1.99	174	∼450	669
*S. cerevisiae*	16	12.07	38.3	5863	1.06	275	∼560	515

rRNA, ribosomal RNA; tRNA, transfer RNA

^*^Genome data sources are provided in Supplementary Table 2.

^†^Supplementary Note 3.

^‡^Total available from NCBI database (accessed 26 March 2015) though 5,735 are indicated in ref. 18.
