# Supplementary material for: Genome analysis of three Pneumocystis species reveals adaptation mechanisms to life exclusively in mammalian hosts
Source: Nat Commun. 2016 Feb 22;7:10740. doi: 10.1038/ncomms10740 (PMC4764891; doi:10.1038/ncomms10740)
Supplement: Supplementary Information — Supplementary Figures 1-15, Supplementary Tables 1-5, Supplementary Notes 1-7, Supplementary Methods and Supplementary References [file ncomms10740-s1.pdf]

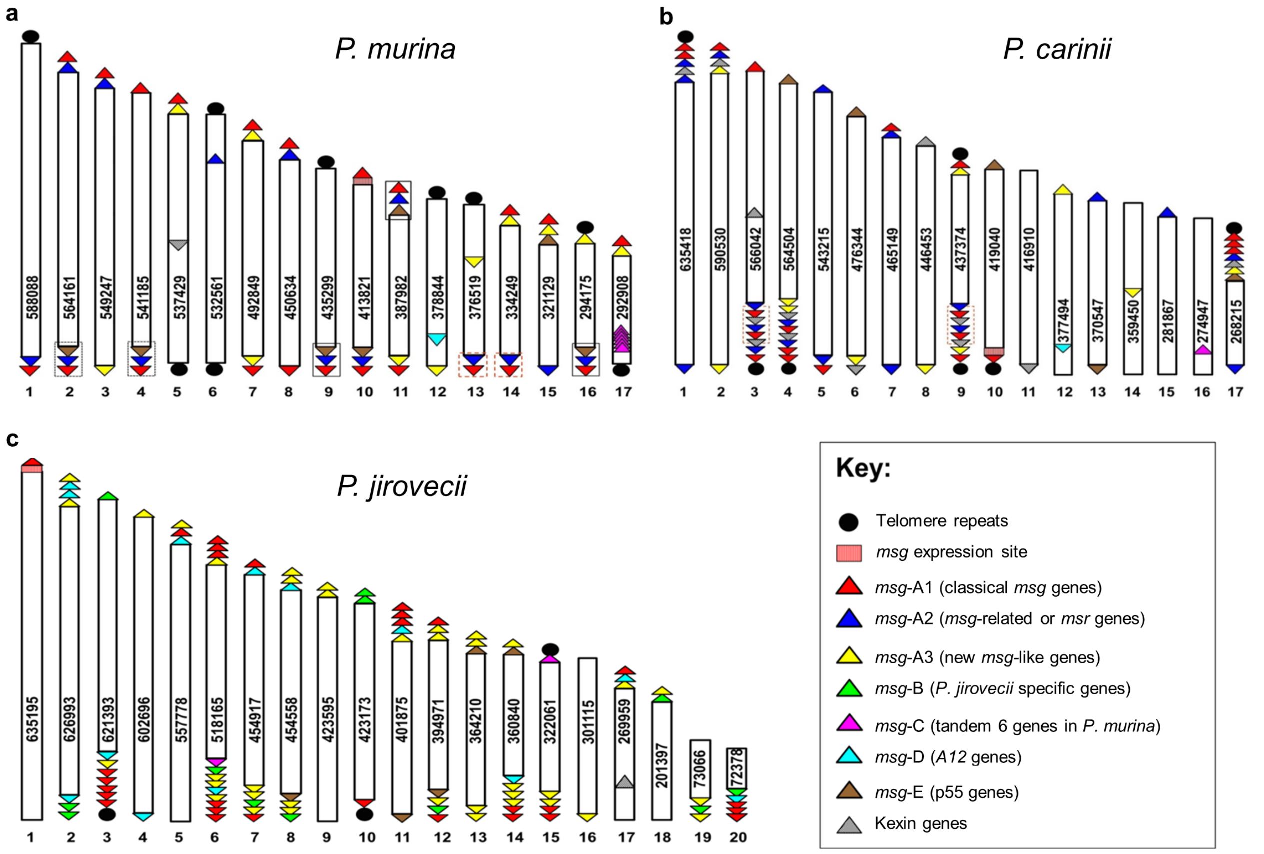

Supplementary Figure 1

Schematic diagrams of three *Pneumocystis* genome assemblies.

Numbers at the bottom in each panel represent the scaffold identification numbers. Numbers inside each bar represent the scaffold length (bp). Duplicated gene clusters in the subtelomeric regions are indicated by boxes with dashed or solid lines, including the 2-gene cluster found in scaffolds 13 and 14, and the 3-gene clusters found in scaffolds 2 and 4, and in scaffolds 9, 11 and 16 in *P. murina*; and the 6-gene cluster found in scaffolds 3 and 9 in *P. carinii*. Features are representative of the chromosome organization and not always drawn to scale. For all three *Pneumocystis* species, all scaffolds are continuous, without gaps or ambiguous bases. Except as noted below, all scaffolds terminate at each end with either telomere/subtelomere repeats or sequences known to reside at subtelomeric regions, including *msg* (all 3 species) or kexin (*P. carinii*) gene family members. Five scaffolds of *P. carinii* and 8 scaffolds of *P. jirovecii* do not terminate with these sequences, and may be linked to some of the small contigs that contain only *msg* sequences (Supplementary Data 1) or kexin sequences (Supplementary Data 3).

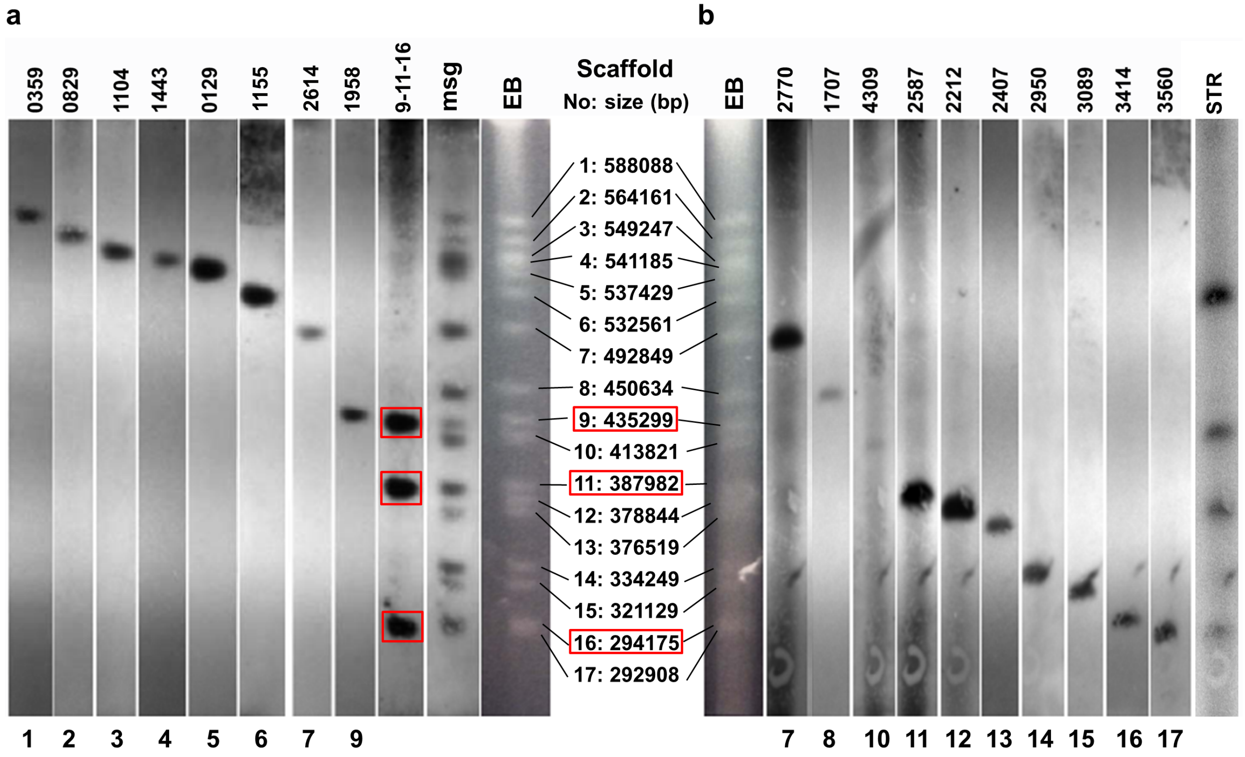

Supplementary Figure 2.

Validation of *P. murina* genome assembly by CHEF and Southern blotting.

(**a** and **b**) show consecutive hybridizations with two different blots. Lane EB, agarose gel stained by ethidium bromide. Probes from single-copy genes specific for each scaffold are indicated by a 4-digit number above each lane, representing the last four digits of the gene ID (for example, 0359 for gene PNEG_00359). The number at the bottom in each panel represents the scaffold number. When using these probes, only a single band was detected for each probe, with a size expected for the corresponding scaffold. When the scaffolds were ordered by length, they were all consistent with the order of chromosomes by length. The hybridization bands for two sets of scaffolds (3 and 4, and 16 and 17) were not well separated but could be distinguished by overlapping developed X-ray films. Probe 9-11-16 contains a DNA fragment shared among three scaffolds (nos. 9, 11 and 16 boxed in red). Probe *msg* contains the putative conserved recombination junction element (CRJE), which is highly conserved among classical *msg*-A1 members but absent in other *msg* members. Signals of hybridization with the *msg* probe were detected on all chromosomes except for chromosomes (or scaffolds) 6 and 12, which are the only two scaffolds without *msg*-A1 sequences. In contrast to a previous CHEF study[1] in which the kexin gene was located on chromosome 6 which did not hybridize with an *msg* probe, we found it on scaffold/chromosome 5 (by sequencing as well as CHEF hybridization using probe 0129), which also showed strong hybridization with the *msg* probe. These observations suggest a possible misalignment of the DNA band in the previous report, or alternatively *P. murina* strain variation. Probe STR contains a subtelomeric repeat sequence from the 5’ end of scaffold 9 as shown in Supplementary Fig. 3a.

a

Scaffold 6, partial 5’ end

TAAGCCTAAGCCTAAGCCTAAGCCTAAGCCTAAGCCTAAGCCTAAGCCTAAGCCTAAGCCTAAGCCTAAGCCTAAGCCTAAGCCTAAGCCTAAGCC
TAAGCCTAAGCCTAAGCCTAAGCCTAAGCCTAAGCCTAAGCCTAAGCCTAAGCCTAAGCCTAAGCCTAAGCCTAAGCCTAAGCCTAAGCCTAAGCC
TAAGCCTAAGCCTAAGCCTAAGCCTAAGCCTAAGCCTAAGCCTAAGCCTAAGCCTAAGCCTAAGCCTAAGCCTAAGCATAAGCATAAGCATAAGCA
TAAGCATAAGCATAAGCATAAGCATAAGCATAAGCATAAGCATAAGCATAAGCATAAGCATAAGCATAAGCATAAGCATAAGCATAAGCATAAGCA
TAAGCATAAGCATAAGCATAAGCCTAAGCCTACGCTGTTAACCCTAATGTGCTTATAATGAAAATT

Scaffold 9, partial 5’ end, used as template to prepare the hybridization probe for subtelomeric repeats (Supplementary Fig. 2, lane STR)

CCCTAACCCTAACCCTAACCCTAACCCTAACCCTAACCCTAACCCTAACCCTAAGCCTAAGCCTAAGCCTAAGCCTAAGCCTAAGCCTAAGCCTAA
GCCTAAGCCTAAGCCTAAGCCTAAGCCTAAGCCTAAGCCTAAGCATAAGCCTAAGCATAAGCATAAGCATAAGCATAAGCCTAACAATAATAATAA
TAATAATCAAAAAATACAAGATTAAATAATTAT

Scaffold 12, partial 5’ end

GCCTAAGCCTAAGCCTAAGCCTAAGCCTAAGCCTAAGCCTAAGCCTAAGCCTAAGCCTAAGCCTAAGCCTAAGCCTAAGCCTAAGCCTAAGCCTAA
GCCTAAGCCTAAGCCTAAGCCTAAGCCTACGCCTAAGCCTACGCCTAAGCCTACGCATAAGCCTACGCATAAGCCTAAGCCTAAGCCTACGCCTAA
GCCTACGCCTAAGCCTACGCATAAGCCTACGCATAAGCCTACGCATAAGCCTACGCATAAGCCTACGCATAAGCCTACGCATAAGCCTACGCATAA
GCCTACGCATAAGCCTACGCATAAGCCTACGCATAAGCCTACGCATAAGCCTACGCATAAGCCTACGCATAAGCCTGTGCAGTTTGA

Scaffold 17, partial 3’ end

TGTTAATGTCATTTTTTTTTAAATTGATGAATAATTTAGTGACGAAACAAGGCGTAGGCGTAGGCGTAGGCTTAGGCGTAGGCGTAGGCGTAGGCG
TAGGCTTAGGCGTAGGCTTAGGCTTAGGCGTAGGCTTAGGCTTAGGCGTAGGCTTAGGCTTAGGCGTAGGCGTAGGCTTAGGCGTAGGCTTAGGCT
TAGGCGTAGGCTTAGGCTTAGGCGTAGGCTTAGGCTTAGGCTTAGGCTTAGGCTTAGGCTTAGGCTTAGGCTTTAGGCTTAGG

b

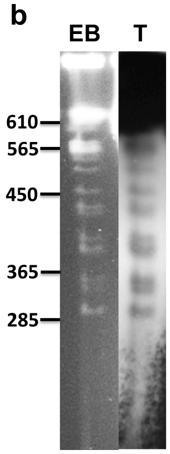

Supplementary Figure 3

Subtelomeric and telomeric repeats in *P. murina*.

(**a**) Subtelomeric repeats in four scaffolds of *P. murina*. Sequences shown include only the portion at the 5’ or 3’ end of the scaffold. Different repeat units are indicated by different colors. (**b**) Detection of telomeric repeats by chromosome hybridization. Location and size (kb) of the markers are indicated at the left. Lane EB, ethidium bromide stained *P. murina* chromosomes resolved by CHEF. Lane T, chromosomes detected by a synthesized oligonucleotide Telom.r4 containing 9 copies of TTAGGG (Supplementary Data 27). Several large chromosomes of *P. murina,* compacted on the top, cannot be visualized due to hybridization of the probe to fragmented host (mouse) telomeres, which have the same repeat sequence.

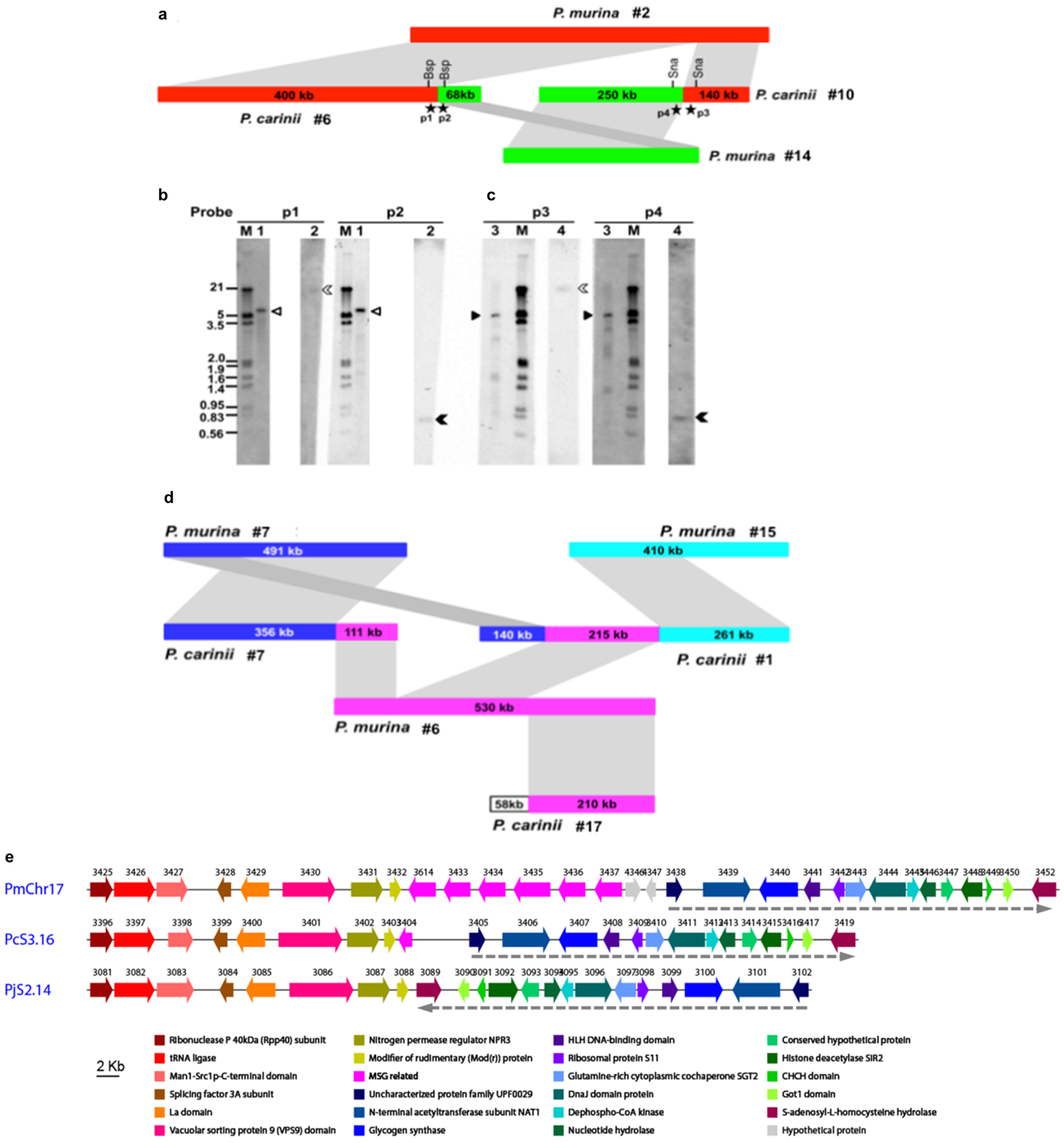

Supplementary Figure 4

Chromosomal rearrangements among *Pneumocystis* genomes.

(a) Schematic representation of two *P. murina* scaffolds (2 and 14) and two *P. carinii* scaffolds (6 and 10). Homologous regions between *P. murina* and *P. carinii* scaffolds are indicated in identical colors, and are connected by the grey shading. Bsp and Sna represent restriction

enzyme Bsp1286I and SnaBI, respectively, used to digest *P. carinii* genomic DNA samples. The locations of four probes (p1, p2, p3 and p4) are marked with stars. **(b)** Hybridization with probes p1 and p2 to confirm rearrangement in *P. carinii* scaffold 6. Lane 1, *P. carinii* genomic DNA digested with Bsp1286I. Lane 2, CHEF blot of *P. murina* chromosomes. **(c)** Hybridization with probes p3 and p4 to confirm rearrangement in *P. carinii* scaffold 10. Lane 3, *P. carinii* genomic DNA sample digested with SnaBI. Lane 4, CHEF blot of *P. murina* chromosomes. Lane M, size markers with DNA sizes (kb) given on the left of panel **b**. Solid and open triangles indicate the DNA band detected in *P. carinii.* Solid and open chevrons indicate the DNA band detected in *P. murina.* Hybridization to the *P. murina* blots is weaker because the probes utilized *P. carinii* sequences, which are similar but not identical to the *P. murina* sequences. **(d)** Schematic representation of three *P. murina* scaffolds (6, 7 and 15) and three *P. carinii* scaffolds (1, 7 and 17). Homologous regions between *P. murina* and *P. carinii* scaffolds are indicated in identical colors, and are connected by the grey shading. All rearrangements were confirmed by hybridization (data not shown). **(e)** An example of a genomic region showing gene duplication or deletion and chromosomal rearrangement among three *Pneumocystis* species. A tandem 6-gene cluster (shown in purple arrows) is identified in the *P. murina* chromosome 17 (PmChr17) and belongs to the *msg*-C family (Fig. 3). Only one partial copy of this family is present in *P. carinii* in scaffold 16 (PcS3.16) in the homologous region while two orthologs in *P. jirovecii* are located in two different scaffolds (not shown). The regions flanking the 6-gene cluster of *P. murina* are conserved in all three species, except that *P. jirovecii* (PjS2.14) has an inversion (from genes T551_03089 to T551_3102) relative to the right side of the *P. murina* tandem cluster, as indicated by the dashed grey line.

a

	Sequence read depth	
	rDNA	Whole genome
P. murina	152	190
P. carinii	224	203
P. jirovecii	398	275

b

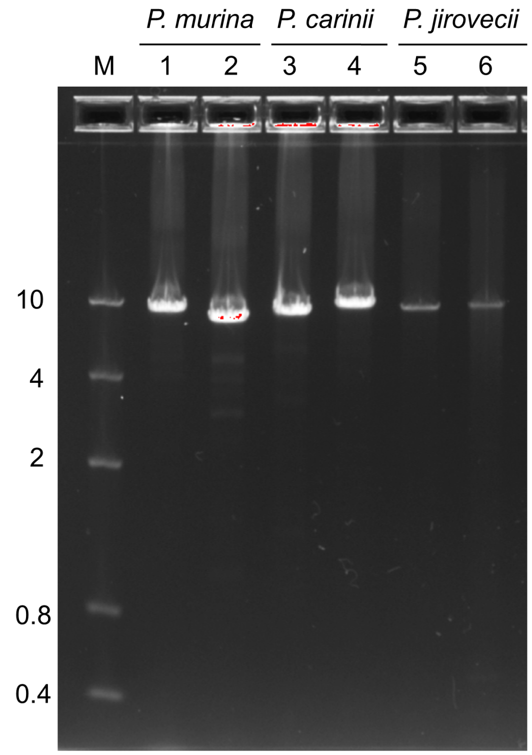

Supplementary Figure 5

Determination of *Pneumocystis* rDNA copy number.

(a) Illumina sequence read depth for the rDNA locus in each *Pneumocystis* species. The Illumina reads used for this analysis are available from the NCBI Sequence Read Archive with accession SRR770457 for *P. murina* B123, SRR1043727 for *P. carinii* B80 and SRR1043749 for *P. jirovecii* RU7. The read depth was determined by the number of total aligned reads multiplied by the read length (101 base) divided by the length of the rDNA locus or whole genome. While there was *S. cerevisiae* rDNA sequence contamination in *P. murina* and *P. jirovecii*, only reads matching these two species at high identity were used for this calculation. **(b)** Analysis of whole rDNA PCR products in 1.2% E-gel (Invitrogen). Lane M, DNA size marker in kb. Primer pair used for each reaction is as follows: Lane 1, Pm2762.r1 and Pm2765.r1; lane 2, Pm18S.f2 and Pm2765.r1; Lane 3, Pc.rDNA.f2 and Pc.rDNA.r1; Lane 4, Pm2762.r1 and Pc.rDNA.r1; Lane 5, Pj.rDNA.f2 and Pj601.r1; Lane 6, Pj.rDNA.f2 and Pj601.r2. Primer sequences are provided in Supplementary Data 27. All primers are located outside the rDNA locus. Partial sequencing of each PCR product confirmed sequence identity. Both the PCR analysis and Illumina read depth support a single-copy rDNA locus.

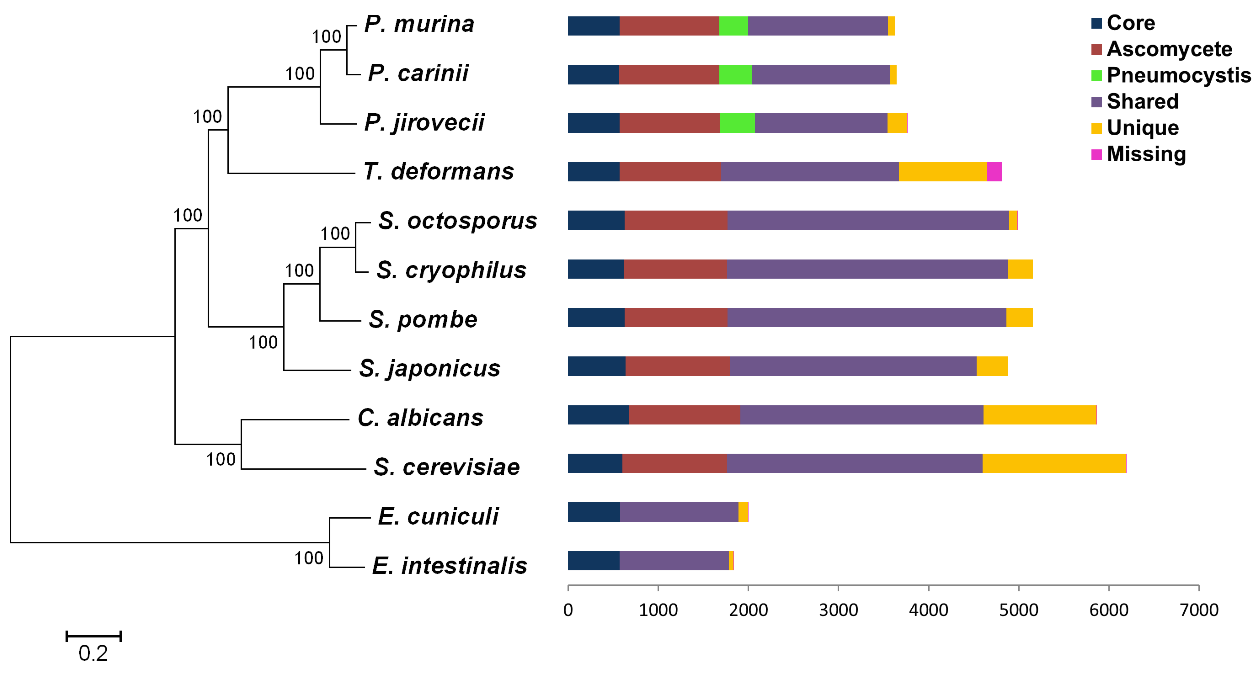

Supplementary Figure 6

Phylogenetic relationship and ortholog conservation for *Pneumocystis* and related fungi. Phylogenetic relationship is inferred from 413 single copy core orthologs. Ortholog conservation patterns highlighted include: core orthologs found in all genomes ('Core'); Ascomycete specific found in all Ascomycetes but not in the Microsporidia ('Ascomycete'); *Pneumocystis* specific; 'Missing', found in all other genomes but absent in one genome; 'Shared', present in any two or more genomes; and 'Unique', found in only one genome. The scale at the bottom indicates the number of orthologous genes.

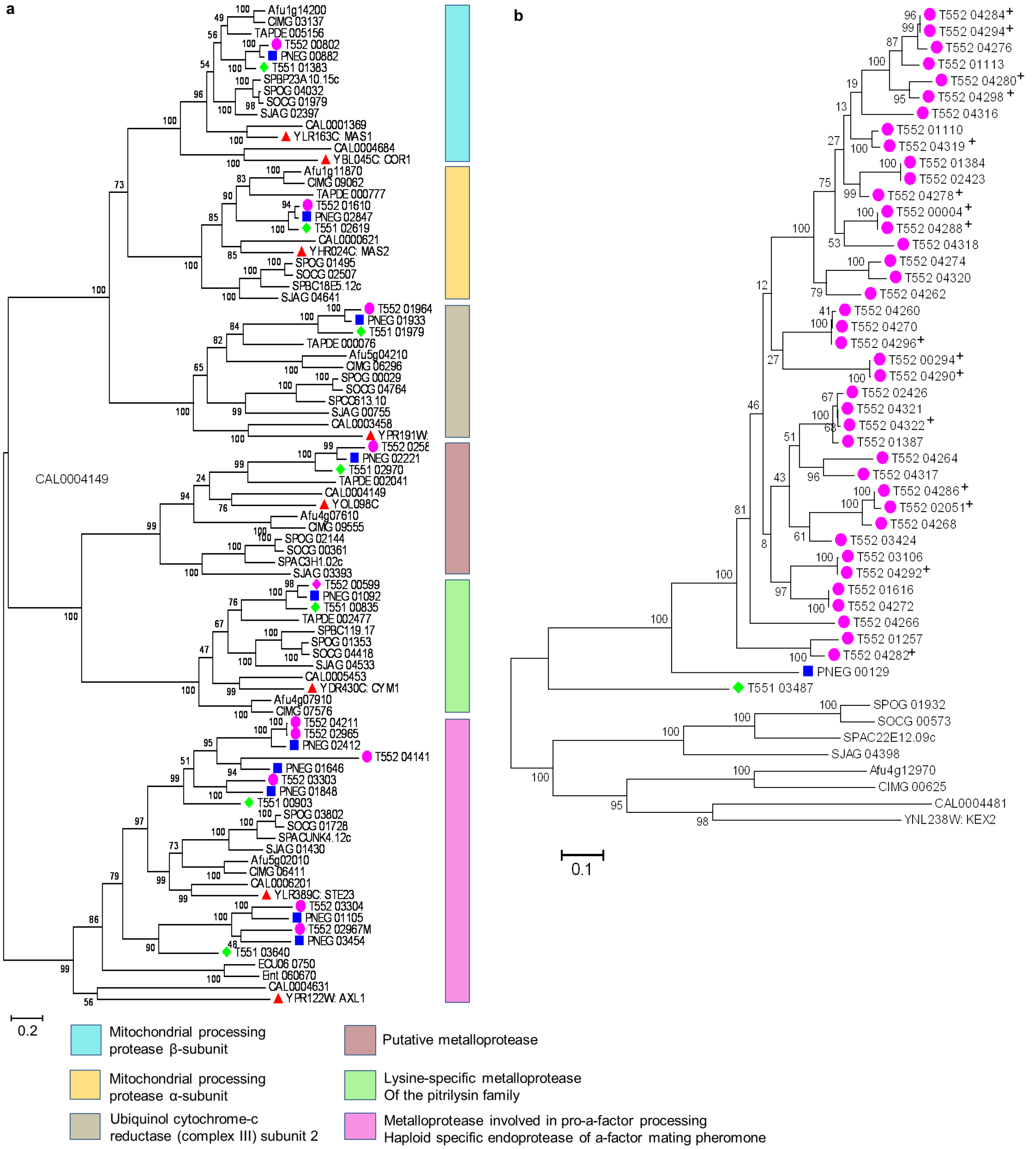

Supplementary Figure 7

Expansion of the M16 and kexin peptidase families in *Pneumocystis*.

(a) Phylogenetic tree of M16 peptidases built using all genes with hits to Pfam domains PF05193 and PF00675, showing that most of the ortholog groups (indicated by the colored vertical bars) are conserved in most of the analyzed species, and most species have a single

copy in each ortholog cluster, except as noted below. *Pneumocystis* species have expansions of the ortholog group (at the bottom) that includes *S. cerevisiae* genes Ste23 (YLR389C) and Axl1 (YPR122W). *P. jirovecii* has two copies, *P. murina* has five copies and *P. carinii* has six copies. The function of each gene family is based on the *S. cerevisiae* genes (red triangles) and indicated at the bottom. (**b**) Phylogenetic tree of kexin genes. A total of 40 kexin genes are present in *P. carinii* with 13 of them mapped to 9 scaffolds (Supplementary Fig. 1b) and the remaining 27 present in short contigs containing either a kexin gene alone or a tandem array of kexin and *msg* genes (several of them are identical but linked to unique *msg* genes, thus defined as separate copies). All other species shown have only one kexin gene per genome, including *P. murina*, *P. jirovecii*, *S. japonicus* (SJAG_04398), *S. pombe* (SPAC22E12.09), *S. cryophilus* (SPOG_01932), *S. octosporus* (SOCG_00573), *C. immitis* (CIMG_00625), *A. fumigatus* (AFUA_4G12970), *C. albicans* (CAL0004481) and *S. cerevisiae* (YNL238W). Genes in *Pneumocystis* are indicated by blue squares for *P. murina*, pink circles for *P. carinii* and green diamonds for *P. jirovecii.* The presence of a predicted GPI anchor is indicated by '+' in the end of the gene ID.

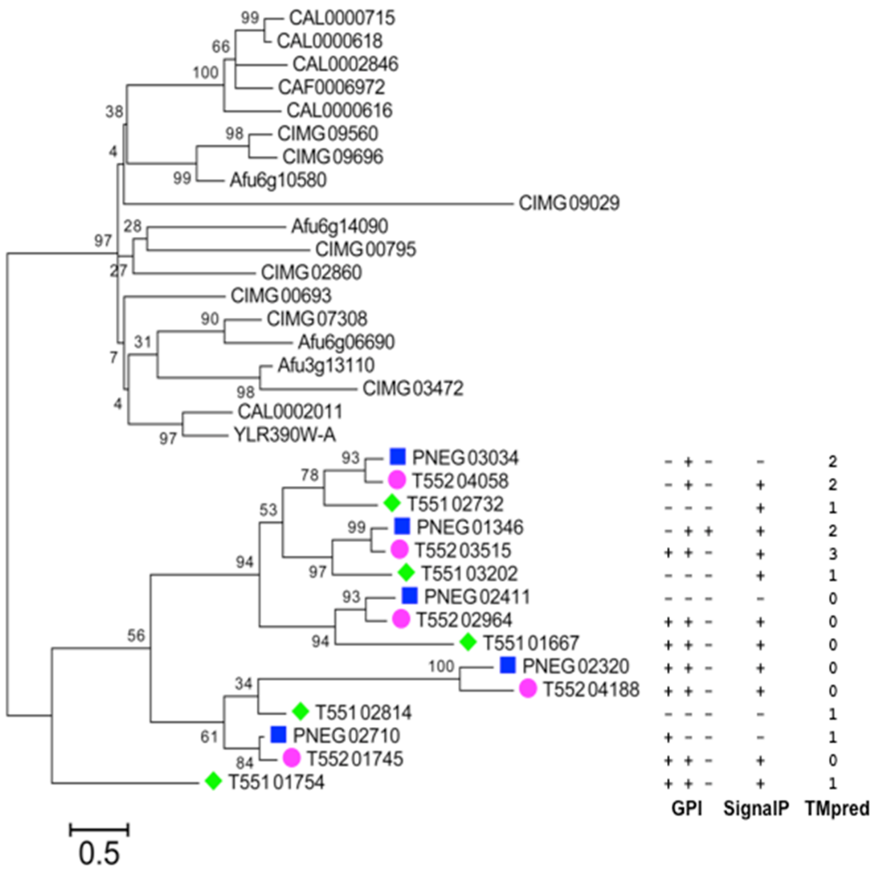

Supplementary Figure 8

Fungal specific cysteine-rich CFEM domain in *Pneumocystis* and related fungi.

The tree includes all *Pneumocystis* genes containing a Pfam PF05730 domain. Each *Pneumocystis* species (blue squares for *P. murina*, pink circles for *P. carinii* and green diamonds for *P. jirovecii*) has 5 members of the CFEM domain-containing gene family (two to three were not included in Fig. 2 due to a higher homology cut-off level used in Fig. 2); each gene has one to six CFEM domains. Other pathogenic species also have expansions of this family, including *A. fumigatus* (4 copies with "Afu" as the prefix in the gene ID), *C. immitis* (8 copies with "CIMG" as the prefix), and *C. albicans* (6 copies with "CAL" or "CAF" as the prefix). *S. cerevisiae* has only one gene (YLR390W-A) and *Schizosaccharomyces* species do not have any. GPI anchor was predicted using the PredGPI (http://gpcr.biocomp.unibo.it/predgpi/pred.htm), big-PI (http://mendel.imp.ac.at/gpi/fungi_server.html) and KohGPI (http://gpi.unibe.ch/) programs, with + and – indicating the presence or absence of a GPI anchor for each method, respectively. Signal peptide was predicted using the SignalP 4.1 server[2]. Transmembrane domains were predicted using the TMpred server (http://www.ch.embnet.org/software/TMPRED_form.html); the number of transmembrane domains is indicated. In *C. albicans*, CFEM proteins are involved in scavenging iron from heme and hemoglobin[3,4].

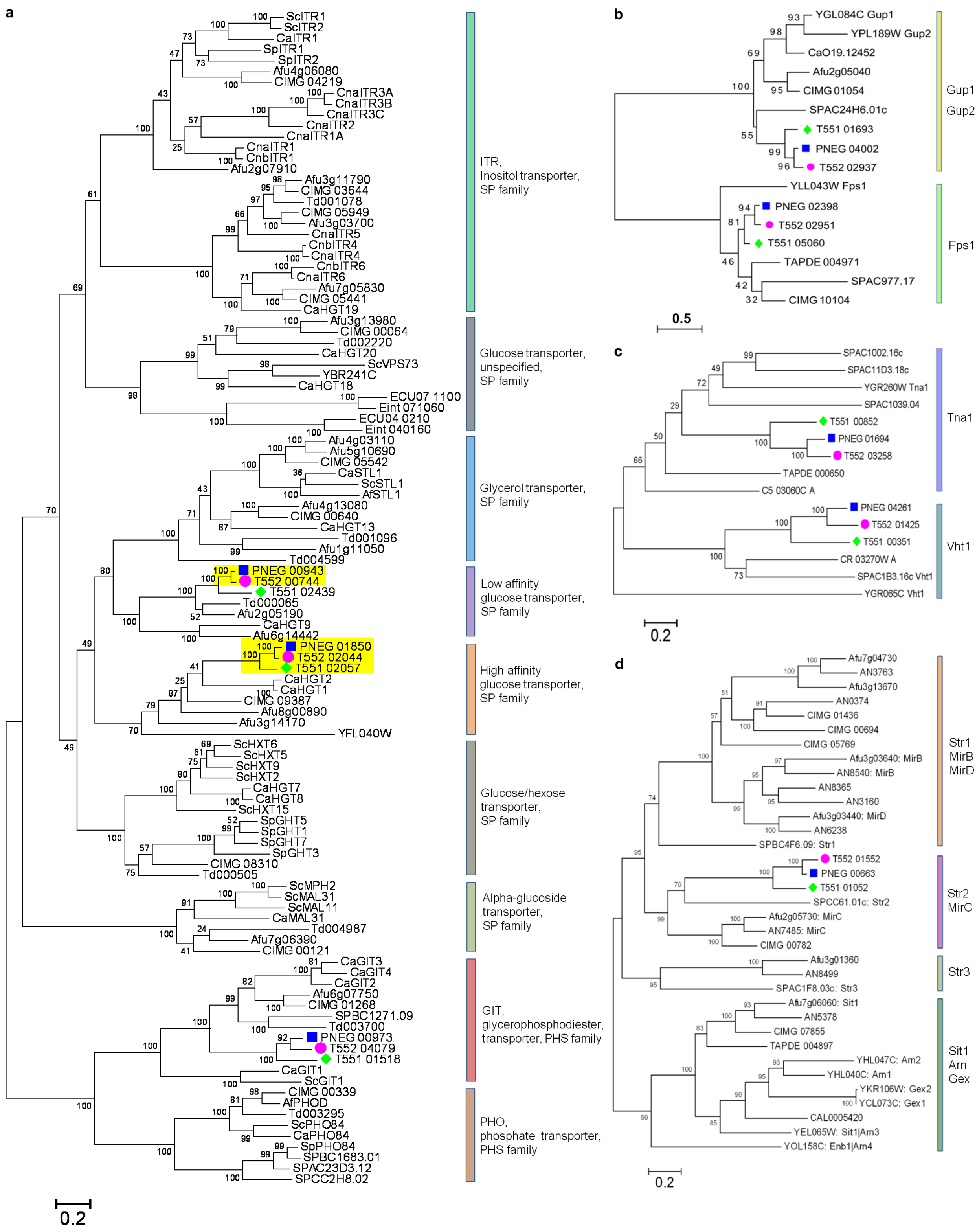

Supplementary Figure 9

Phylogenetic analysis of transporters in *Pneumocystis* and related fungi.

(**a**) Sugar transporters including different SP and PHS family members. The genes previously suggested to be inositol transporters in *Pneumocystis*[5] are highlighted in yellow; they are more closely related to glucose transporters in this analysis. (**b**) Glycerol exporters (Gup1 and Gup2) and importer (Fps1). (**c**) Nicotinic acid (Tna1) and biotin (Vht1) transporters. (**d**) Siderophore-iron transporters. Each *Pneumocystis* species has a homolog to the yeast siderophore-iron transporter Str2 and MirC. All gene names starting with Sc refer to genes in *S. cerevisiae*. For all other gene names, see the Methods. Genes present in *Pneumocystis* are indicated by blue squares for *P. murina,* pink circles for *P. carinii,* and green diamonds for *P. jirovecii*.

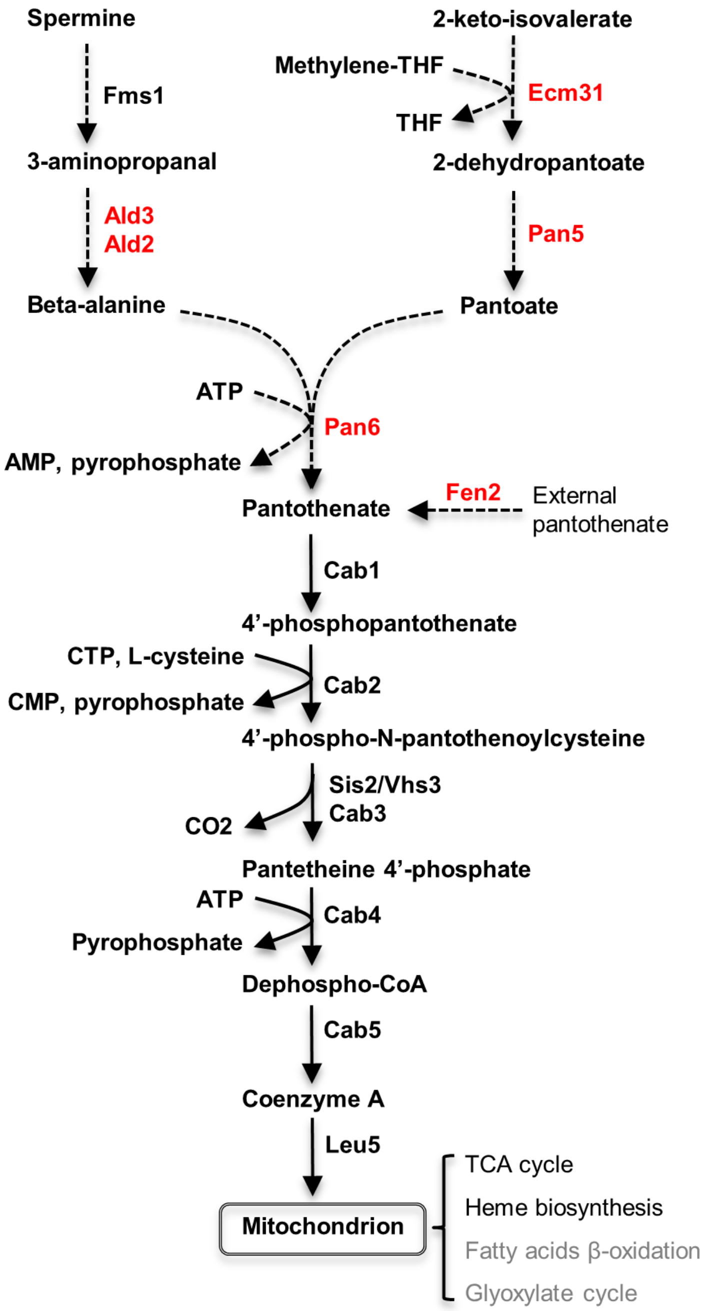

Supplementary Figure 10

Loss of CoA synthesis pathway in *Pneumocystis*.

Highlighted in red are enzymes and transporter (Fen2) absent in all three *Pneumocystis* species. CoA is involved in TCA cycle, heme biosynthesis, fatty acid β-oxidation and glyoxylate cycle in other fungi though the latter two pathways (indicated in grey) are lost in *Pneumocystis*.

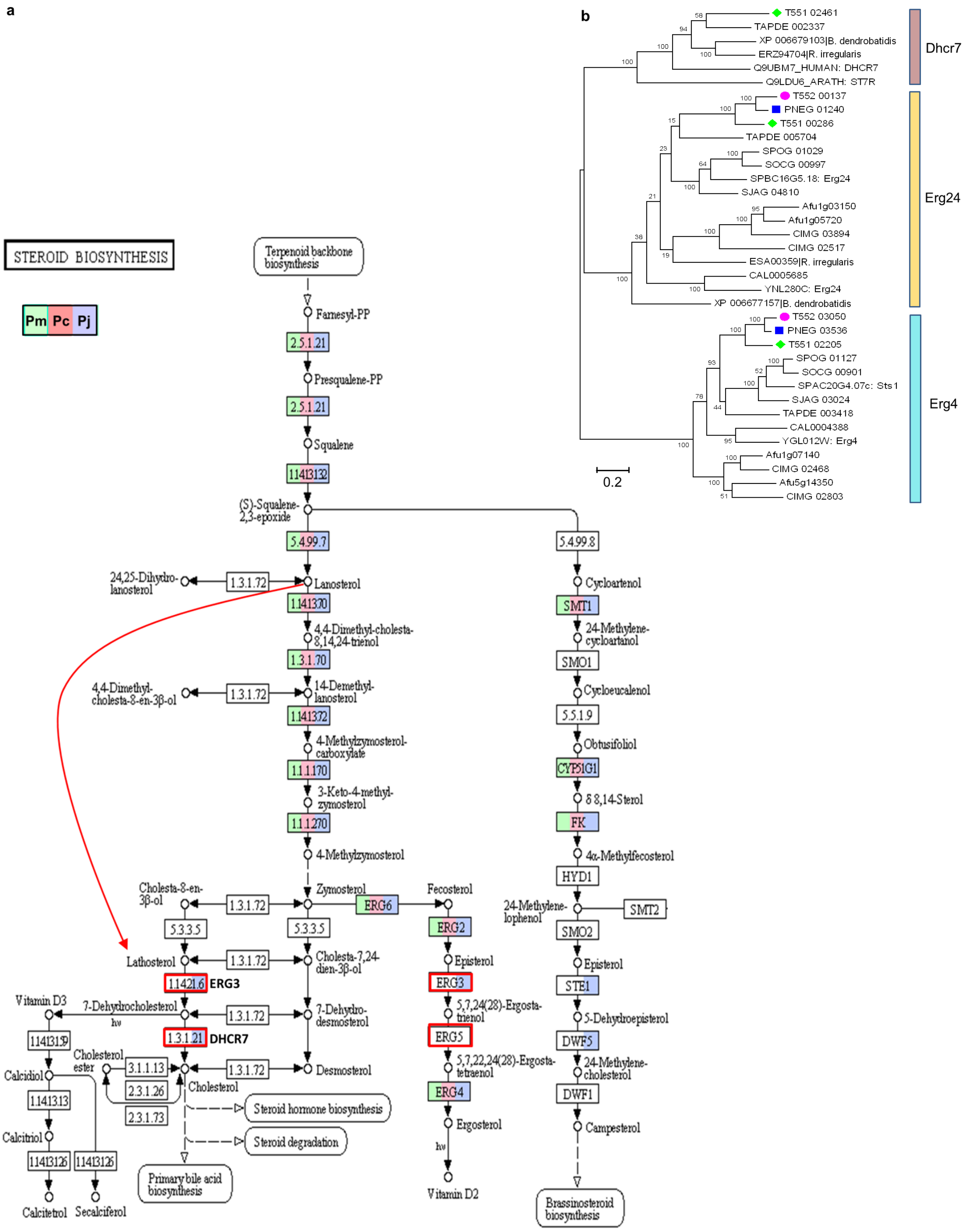

Supplementary Figure 11

Pneumocystis **sterol biosynthesis pathways.**

(**a**) KEGG map created using the data in Supplementary Data 12. The enzymes present in *P. murina* (Pm), *P. carinii* (Pc), and *P. jirovecii* (Pj) are highlighted in colors as indicated by the key. Absence of color indicates a loss of enzyme. Of note, ERG5 (boxed in red) is absent in all three *Pneumocystis* species while ERG3 and DHCR7 (boxed in red) are absent in both *P. murina* and *P. carinii* but present in *P. jirovecii*. (**b**) Phylogenetic analysis of three key enzymes involved in cholesterol synthesis. Two enzymes (Erg24 and Erg4 like) are conserved in *P. jirovecii* (green diamonds), *P. murina* (blue squares), *P. carinii* (pink circles) and other fungi; the third enzyme (Dhcr7) is found only in limited fungal species, including *P. jirovecii* and *T. deformans*, as well as humans (Q9UBM7) and *Arabidopsis thaliana* (Q9LDU6).

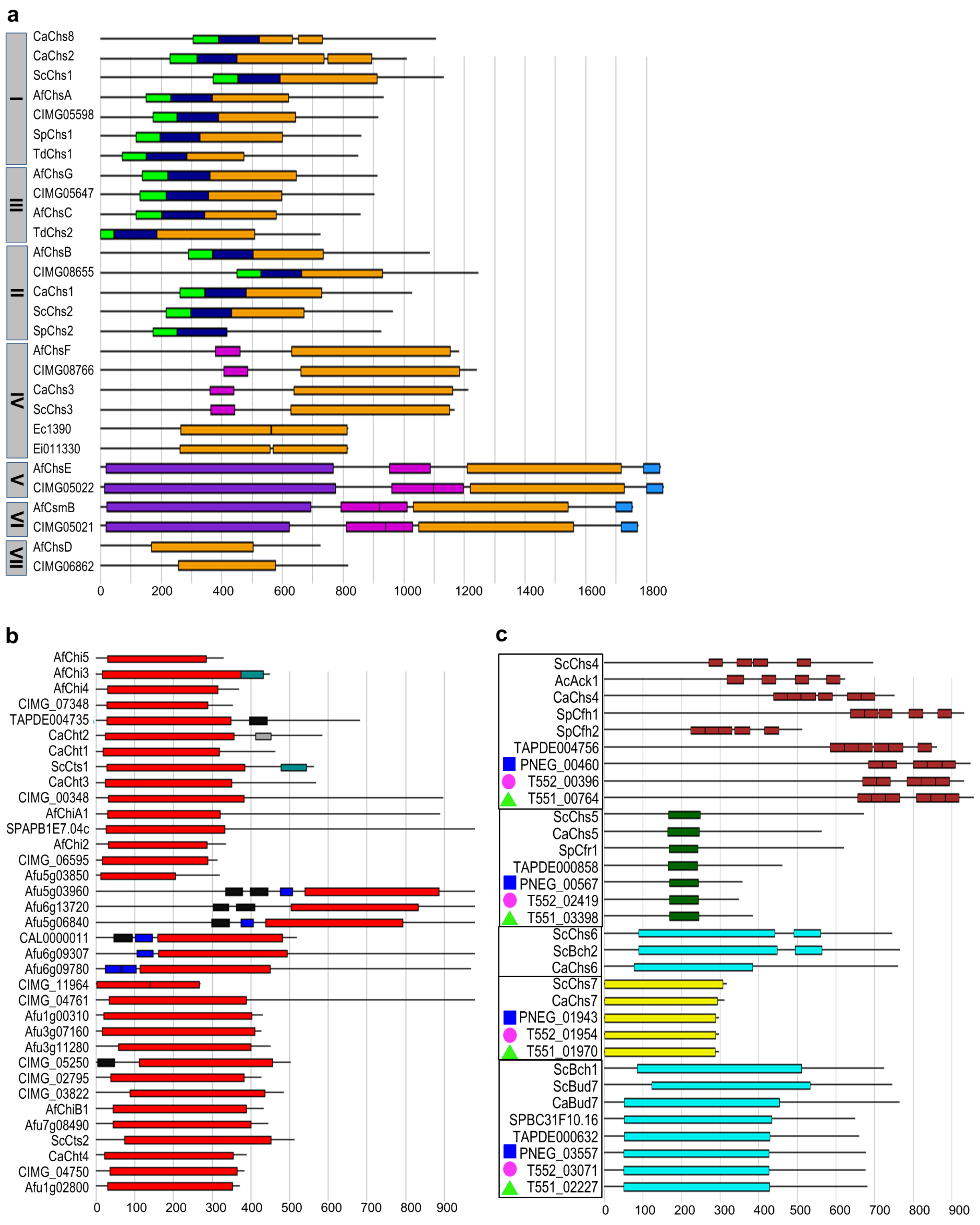

Supplementary Figure 12

Protein domain analysis of chitin synthases (a), chitinases (b) and other accessory proteins (c) involved in chitin metabolism in *Pneumocystis* and other related fungi.

The names of enzymes and proteins follow the standard abbreviated names for *S. cerevisiae* (See more details in the Methods and Supplementary Data 17). Four proteins identified in *Pneumocystis* (c) are indicated by blue squares for *P. murina*, pink circles for *P. carinii* and green diamonds for *P. jirovecii*, including the previously reported PcCh5 gene[6] (identical to T552_02419 in this study); none of the accessory proteins (**c**) contains any domains of chitin synthases (**a**) or chitinases (**b**).

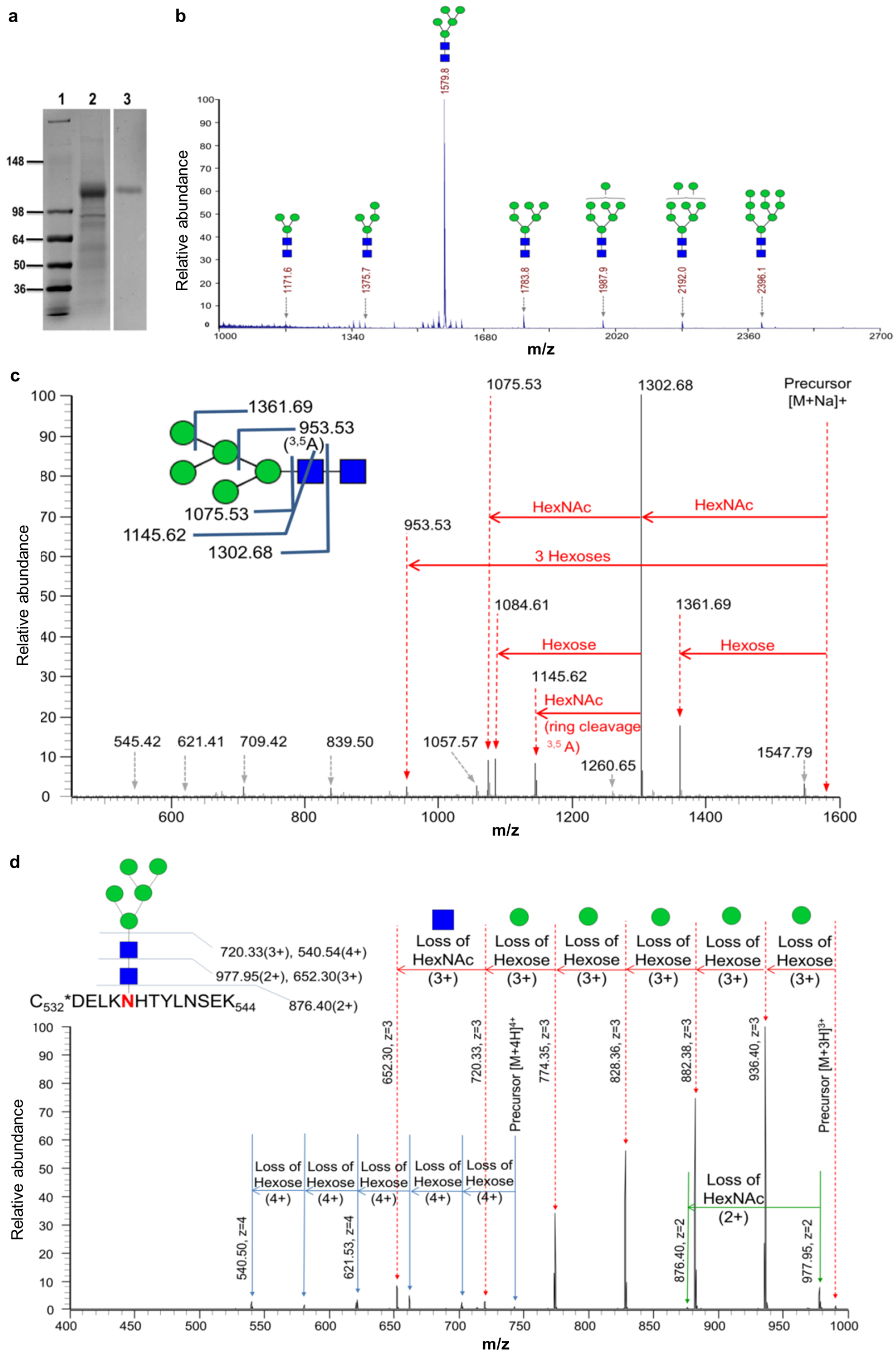

Supplementary Figure 13

Experimental identification of N-linked glycans in *P. carinii* Msg proteins.

(a) Affinity purified *P. carinii* Msg proteins in SDS-PAGE gel stained with Coomassie blue. Lane 1, protein size marker in kDa; lane 2, total protein extract; lane 3, concentrated purified

Msg proteins. **(b)** MALDI/TOF-MS profile of released permethylated N-glycans from purified *P. carinii* Msg proteins. N-glycans were released enzymatically from purified *P. carinii* Msg proteins by PNGase F treatment. The released N-glycans were permethylated and profiled by MALDI/TOF-MS. The Hexose 5 HexNAc2 (M5N2) at m/z 1579.8 was detected as the predominant N-glycan component. Other pauci- and high-mannose type structures were also observed as minor components. Among those N-glycans detected, Hexose 5 HexNAc2 and Hexose6 HexNAc2 were further confirmed to be from *P. carinii* Msg by glycopeptide analysis as shown in Fig. 7, Supplementary Data 21, and panel **d** below. **(c)** MS/MS spectrum of a released permethylated N-glycan from *P. carinii* Msg at m/z 1580, Hexose 5 HexNAc2 (Na+ form, singly charged). Glycan signals detected by MALDI/TOF-MS (**b**) were analyzed by NSI-MSn (LTQ-orbitrap Fusion) for the sequence of the N-linked oligosaccharides. The main fragment ion observed was m/z 1302, b-type fragment ion from neutral loss of the reducing end GlcNAc. A series of neutral losses from the non-reducing end hexoses were observed as minor fragments. The fragmentation pattern was in good agreement with a typical fragment pattern of the Hexose 5 HexNAc2 N-glycan structure. **(d)** MS/MS-collision-induced dissociation (MS/MS-CID) spectrum of N-linked glycopeptides from one Msg isoform (T552_03736) in *P. carinii*. A series of fragment ions due to neutral loss of 1 to 5 hexoses (Δ m/z 54 as triply charged, Δ m/z 40.5 as quadruple charged ions, per hexose), followed by 2 HexNAcs (Δ m/z 101.5 as doubly charged, Δ m/z 68 as triply charged, per HexNAc) were detected. These results, together with the MS/MS-HCD and MS/MS-ETD data for the same Msg isoform presented in Fig. 7c, d, indicate that the Msg isoform carries Hexose 5 HexNAc2 (M5N2) as the main N-linked glycan modification.

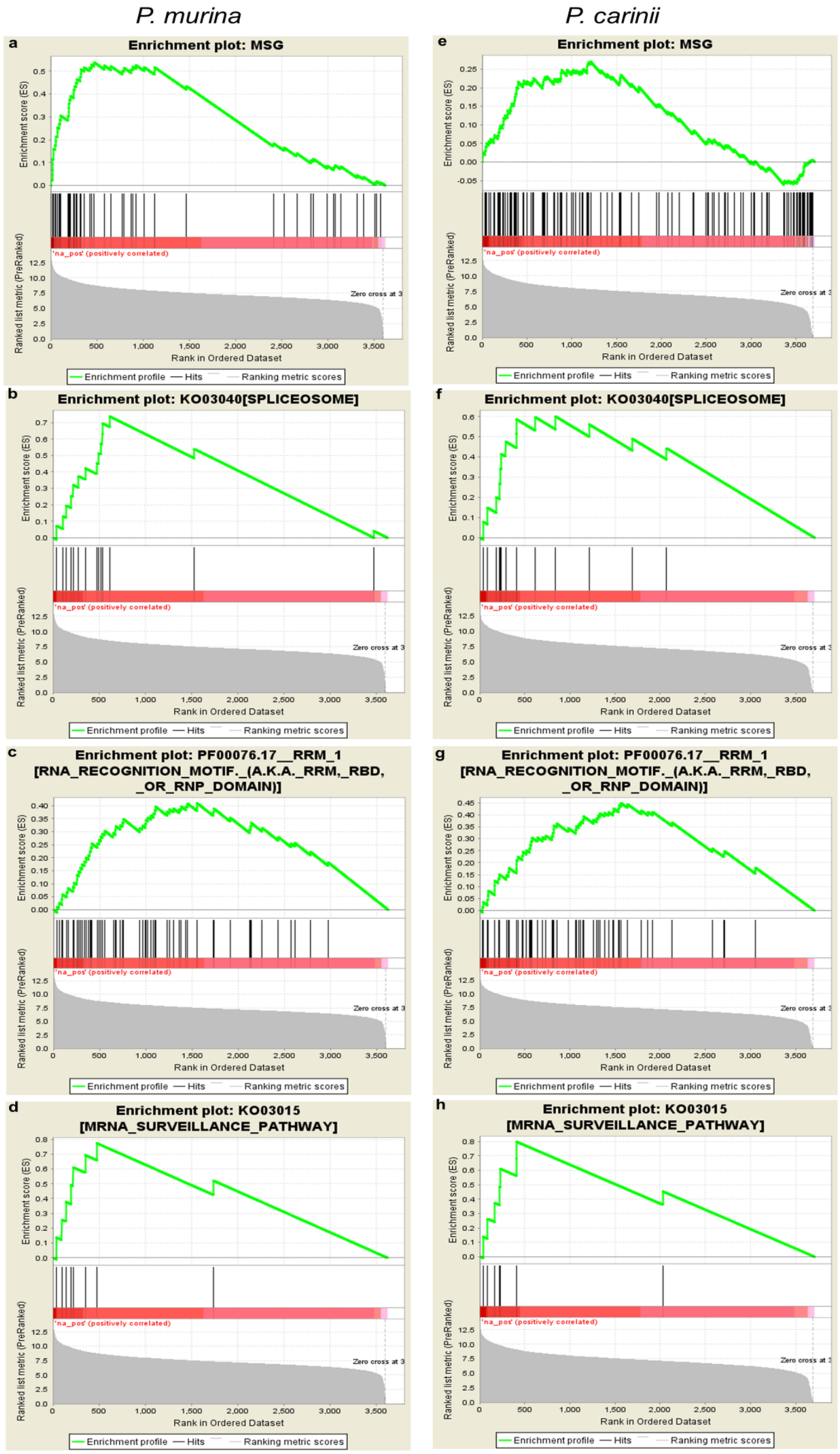

Supplementary Figure 14

***Pneumocystis* gene set expression enrichment plots.**

For both *P. murina* and *P. carinii*, we examined the *in vivo* gene expression for enriched gene sets. These included the Msg (**a**, **e**), spliceosome (**b**, **f**), RNA recognition (**c**, **g**), and mRNA surveillance pathway (**d**, **h**) gene sets. Each panel shows enrichment profile (green line) and individual gene values (black lines) relative to gene expression levels for all genes, shown in grey (genes ordered from high to low expression values measured by $\log_2$(FPKM)).

```
         10        20        30        40        50        60        70        80        90       100
AAGGAGATATACCATGGCAACAACAAATCCCGGCGTTAGTGCTTGGCAGGTTAATACCGCTTATACCGCAGGACAGCTGGTTACATACAATGGCAAAACA
                 M  A  T  T  N  P  G  V  S  A  W  Q  V  N  T  A  Y  T  A  G  Q  L  V  T  Y  N  G  K  T

        110       120       130       140       150       160       170       180       190       200
TATAAATGCCTGCAGCCACACACCAGCCTGGCAGGATGGGAACCATCCAACGTGCCAGCACTGTGGCAGCTGCAGTACCCATACGATGTTCCTGACTATG
 Y  K  C  L  Q  P  H  T  S  L  A  G  W  E  P  S  N  V  P  A  L  W  Q  L  Q  Y  P  Y  D  V  P  D  Y

        210       220       230       240       250       260       270       280       290
CGTATCCCTATGACGTCCCGGACTATGCATATCCATATGACGTTCCAGATTACGCTCTCGAGCACCACCACCACCACCACTGAGATCCGGCTGC
A  Y  P  Y  D  V  P  D  Y  A  Y  P  Y  D  V  P  D  Y  A  L  E  H  H  H  H  H  H  *
```

Supplementary Figure 15

Nucleotide and deduced amino acid sequences of construct for expression of chitin binding domain (CBD) of *Bacillus circulans* chitinase A1 gene.

The nucleotide sequence optimized for bacterial expression is shown. Nco1 and Xho1 restriction sites are underlined. Amino acid sequences corresponding to HA tags are double underlined. The sequences upstream of Nco1 and downstream of Xho1 restriction sites are from the vector, pET28b (Novagen).

Supplementary Table 1. Statistics of different *Pneumocystis* genome assemblies.

	P. murina	*P. carinii*	*P. jirovecii*	*P. carinii*	*P. jirovecii*
Source	This study	This study	This study	Slaven *et al.*[7]	Cisse *et al.*[8]
Assembly size (Mb)	7.5	7.7	8.4	6.3	8.1
Scaffolds or contigs (n)	17[a]	17[a]	20[a]	4,272	358
N50 (kb)	491.3	465.2	454.6	2.2[b]	41.6
GC content (%)	26.9	27.6	28.8	31.1	29.1
Protein-coding genes (n)	3,623	3,646	3,761	4,591[b]	3,282
Protein length (aa, mean)	460	465	474	291	409
Protein length (aa, range)	46-5,059	46-5,030	47-5,097	10-3,393	14-5,307
rRNA genes (n)	5	5	5	2	2
tRNA genes (n)	47	45	46	20[b]	36
Exons (n)	22,032	21,759	21,720	n/a	14,422

[a] Not including short contigs for *msg* genes. No full-length *msg* genes were included in previously reported assemblies[7,8].

[b] Updated by Cisse *et al* [8].

Supplementary Table 2. Genome data sources for fungal species compared in this study.

Genome	Reference	Web link
P. murina	This work	http://www.ncbi.nlm.nih.gov/bioproject/70803
P. carinii	This work	http://www.ncbi.nlm.nih.gov/bioproject/?term= PRJNA223511
P. jirovecii	This work	http://www.ncbi.nlm.nih.gov/bioproject/?term= PRJNA223510
T. deformans	Cisse *et al.*[9]	http://www.ncbi.nlm.nih.gov/genome/11847? genome_assembly_id=40296
S. pombe	Wood *et al.*[10]	http://www.pombase.org
S. octosporus	Rhind *et al.*[11]	http://www.ncbi.nlm.nih.gov/bioproject/13639
S. cryophilus	Rhind *et al.*[11]	http://www.ncbi.nlm.nih.gov/bioproject/38373
S. japonicus	Rhind *et al.*[11]	http://www.ncbi.nlm.nih.gov/bioproject/13
S. cerevisiae	Cherry *et al.*[12]	http://www.yeastgenome.org
C. albicans	van het Hoog *et al.*[13]	http://www.candidagenome.org
E. cuniculi	Katinka *et al.*[14]	http://microsporidiadb.org
E. intestinalis	Corradi *et al.*[15]	http://microsporidiadb.org

Supplementary Table 3. Summary of the *msg* superfamily identified in three *Pneumocystis* species.

Family	Subfamily	No. of *msg* genes			Conserved domains	Note
		P. murina	*P. carinii*	*P. jirovecii*		
msg-A	*msg*-A1	25	66	80	N1, M1-6, C1-2	Classical *msg* genes with a partial CRJE at the 5' end.
msg-A	*msg*-A2	14	50	0	N1, M1-6, C1-2	*msr* genes with a conserved exon at the 5' end.
msg-A	*msg*-A3	11	18	51	N1, M1-6, C1-2	Lack of a conserved 5' end sequence as in classical *msg* or *msr* genes.
msg-B	n/a	0	0	21	N1, M1, M3	*P. jirovecii* specific genes.
msg-C	n/a	6	1	2	N1, M1, M3	A 6-gene cluster in *P. murina.*
msg-D	n/a	1	1	20	N1, M1-5	A12 related genes.
msg-E	n/a	7	5	5	N1	*p55* related genes.
Total		*64*	*141*	*179*		

Supplementary Table 4. Plasma membrane transporters retained in *P. jirovecii* as potential new drug targets.

P. jirovecii gene ID	*S. cerevisiae* homolog	Substrate	Role in metabolism
T551_02142	Ptr2	Peptide	Amino acid synthesis
T551_02057	Hgt1	Glucose	Energy/nutrient supply
T551_01693	Gup1	Glycerol	Nutrient & osmoregulation
T551_03393	Dnf1	Phosphatidylcholine	Membrane structure
T551_01518	Git1	Glycerophosphodiesters	Membrane structure
T551_04121	Tpo1	Polyamine	Various functions
T551_01296	Thi9	Thiamine or vitamin B1	Various metabolism
T551_00852	Tna1	Nicotinic acid	Various metabolism
T551_01425	Vht1	Biotin or vitamin H	Various metabolism
T551_01052	Sit1	Iron	Iron uptake

Supplementary Table 5. qPCR analysis of genomic DNA in *Pneumocystis* mRNA samples.

Sample Name	DNase treatment	Genomic DNA tested[a]	C_t Median[b]	DNA (ng) used for qPCR
RPc1	Before	*P. carinii, R. norvegicus*	29.8	4.9
RPc1	After	*P. carinii, R. norvegicus*	35.1	1.4
RPc2	Before	*P. carinii, R. norvegicus*	30.5	9.8
RPc2	After	*P. carinii, R. norvegicus*	34.8	1.6
RPc3	Before	*P. carinii, R. norvegicus*	30.2	5.0
RPc3	After	*P. carinii, R. norvegicus*	35.2	1.5
MPm1	Before	*P. murina, M. musculus*	28.6	10.4
MPm1	After	*P. murina, M. musculus*	34.2	1.5
MPm2	Before	*P. murina, M. musculus*	27.4	5.2
MPm2	After	*P. murina, M. musculus*	32.6	1.5
MPm3	Before	*P. murina, M. musculus*	28.2	5.2
MPm3	After	*P. murina, M. musculus*	32.7	1.5
NTC[c]	N/A[d]	*P. carinii*	34.3	0
NTC[c]	N/A[d]	*P. murina*	32.9	0
NTC[c]	N/A[d]	*M. musculus*	34.4	0

No genomic DNA detectable for either fungus or host after DNase treatment based on the Ct Median values compared to those of the NTCs.

[a]Genomic DNA standards were run for all 4 species.

[b]Each C_t Median derived from 3 qPCR reaction wells.

[c]NTC: No template control. NTC for *R. novegicus* not done.

[d]N/A: Not applicable.

Supplementary Notes

Supplementary Note 1: Confirmation of duplicated regions and telomere repeats.

To verify three sets of duplicated gene cassettes in the end of 7 scaffolds in *P. murina* (Supplementary Fig. 1a), we performed contour clamped homogeneous electrical field (CHEF) electrophoresis and hybridization with probes from a region shared within each duplicated set. Two or three bands were detected for each probe, with a size expected for the corresponding scaffolds. An example of these hybridization experiments is shown in Supplementary Fig. 2a. These experiments confirmed that copies of these gene cassettes are present on multiple scaffolds. CHEF electrophoresis and hybridization were also used to verify the telomere/subtelomere repeats in *P. murina*. When using an oligonucleotide probe containing 9 copies of the telomere repeat unit TTAGGG, hybridization signal was detected on all visualized chromosomes (Supplementary Fig. 3b). When using a DNA probe amplified from *P. murina* genomic DNA, which contained a mixture of three different repeat units TTATGC, TTAGGC and TTAGGG (Supplementary Fig. 3a), hybridization occurred predominantly with 4 chromosomes corresponding to scaffolds 6, 9, 12 and 17 (Supplementary Fig. 2b), all of which contained a mixture of 2 or 3 different repeat units with the TTAGGC unit being the most predominant. These experiments suggest that the TTAGGG sequence is the telomere repeat unit present in all *P. murina* chromosomes and that other repeat sequences (TTAGGC, TTATGC and GTAGGC) may be subtelomere repeats present in only 4 chromosomes (Supplementary Fig. 2b).

When using the *P. murina* telomere/subtelomere repeats to blast Illumina raw reads from *P. carinii* and *P. jirovecii*, a large number of reads was found for the repeat unit TTAGGG (linked to *Pneumocystis*-specific sequences) while no reads were found for other repeat units (TTAGGC, TTATGC, and GTAGGC) in either species. These data support TTAGGG as the telomere repeat unit in all three *Pneumocystis* species.

We were unable to perform CHEF studies with *P. carinii* or *P. jirovecii* since CHEF analysis requires fresh, heavily infected lung tissues, which are currently unavailable to us.

Supplementary Note 2: Confirmation of chromosomal rearrangements.

We found rearrangements of ~60-260 kb segments in five *P. carinii* scaffolds compared to the *P. murina* assembly (Fig. 1a; Supplementary Fig. 4). All rearrangements were first

analyzed by PCR using primers covering the rearranged regions. Sequencing of the PCR products showed sequences perfectly consistent with the scaffold assemblies. In addition, we performed Southern blotting analysis using CHEF-separated *P. murina* chromosomes as described above and restriction fragments of *P. carinii* genomic DNA as described in our previous report[16]. Representative hybridization results are shown in Supplementary Fig. 4b, c. All rearrangements were supported by hybridization results.

By contrast, the *P. jirovecii* assembly showed more extensive rearrangements, both inter- and intra-chromosomal, with each of the 17 largest scaffolds (potentially representing chromosomes) mapped to 2-5 different chromosomes of *P. murina*. Four rearranged regions in scaffold 9 were chosen for verification by PCR; all of them were successfully amplified from *P. jirovecii* genomic DNA, and the resulting sequences were perfectly consistent with the scaffold assembly.

Supplementary Note 3: rRNA and tRNA genes.

Consistent with previous studies[17-19], each *Pneumocystis* species has a single rDNA locus (or rRNA operon) consisting of three genes (one copy each) and two internal transcribed spacers (ITS1 and ITS2) in the order of 18S rRNA - ITS1- 5.8S rRNA - ITS2 - 26S rRNA. The rDNA copy number in each genome is supported by the coverage depth of sequence reads (Supplementary Fig. 5a) and the detection of a single band in a CHEF blot hybridized with the rDNA probe 2770 (Supplementary Fig 2b). Furthermore, by PCR targeting a 9-11 kb fragment covering the entire rDNA and a part of its upstream and downstream protein-coding genes in each *Pneumocystis* species, we amplified a single band with the expected size and terminal sequence (Supplementary Fig. 5b). In addition to the rDNA locus, each *Pneumocystis* has two copies of 5S rRNA genes present as a tandem repeat (separated by an intergenic region) in a different chromosome than the rDNA locus.

Comparative analysis indicates *Pneumocystis* have the smallest number of rRNA genes among fungi, similar to *Taphrina deformans*, which is a slow growing fungal pathogen in plants[9] and which also contains only a single rRNA operon[9] and two copies of 5S rRNA genes (identified from the sequences deposited in NCBI database). In contrast, other eukaryotes, including the intracellular Microsporidia[15], as well as many bacteria, contain multiple copies (up to tens of thousands) of rDNA per genome[20,21]. For example, *S. cerevisiae* and *S. pombe* contain

a total of ~560 and ~450 rRNA genes, respectively, based on an estimated 140 copies of the rRNA gene cassette present in the genomes of *S. cerevisiae*[12,22,23] and *S. pombe*[10]; each cassette includes 4 genes for *S. cerevisiae* and 3 genes for *S. pombe*. The total for *S. pombe* also includes 5S rRNA genes that are located outside these cassettes[10] (Table 1).

In each *Pneumocystis* species, a total of 45-47 tRNA genes were identified by tRNAScan-SE[24], similar to that in Microsporidia[15] but much less than that in other fungi and eukaryotes (170-570 copies)[25].

It is generally assumed that rDNA redundancy allows the cell to maintain a functional ribosome in diverse environments and that a higher rDNA copy number allows for an increased rate of rRNA synthesis, thus a higher level of ribosome production and more rapid growth[26]. The reduction to a single rDNA copy in *Pneumocystis* may reflect its genome stability as a result of adaptation to a stable environment[27,28]. The single rDNA copy together with a minimal number of tRNA genes as well as an extremely low GC content in *Pneumocystis* also suggests a slow transcription and translation machinery[21,26,29], which presumably reflects a response to resource availability (e.g. a large amount of energy and resources used for intron processing, Fig. 5). The low-levels of transcription and translation may ultimately lead to a slow growth of *Pneumocystis* organisms as discussed in the main text.

Supplementary Note 4: Major surface glycoprotein (Msg) identification and domain and phylogeny analyses.

Available data suggest that *msg* genes are not expressed unless they are translocated downstream of and in-frame with a unique, single-copy subtelomeric region termed the *msg* expression site or the upstream conserved sequence (UCS)[30-32], which encodes a signal peptide needed to localize Msg to the endoplasmic reticulum. A single organism appears to express only a single Msg isoform at a given time, although multiple isoforms are expressed at the population level in immunosuppressed hosts[33]. Variation of the expressed copy of Msg potentially facilitates evasion of host immune responses[33-35]. The *msg* gene family has been identified in all *Pneumocystis* species surveyed, with an estimated ~30-100 copies per species[1,36,37]. An additional gene family encoding Msg-related proteins (Msr) has been identified only in *P. carinii*, with an estimated 60 members[36,38]. The sequences of *msr* genes are very similar to *msg*

genes, but *msr* genes are not dependent on the UCS for expression; each *msr* gene can potentially be expressed independently.

With near complete genomes for all three species, we identified a large number of *msg*, *msr* and additional related genes, collectively termed the *msg* superfamily, including 64, 141 and 179 members in *P. murina*, *P. carinii* and *P. jirovecii*, respectively (Fig. 3; Supplementary Table 3 and Supplementary Data 1), which represents the largest surface protein family identified to date in the fungal kingdom[34]. Using sequence alignments, we defined nine conserved domains, named N1, M1 to M6, C1, and C2 (Fig. 3; Supplementary Table 3 and Supplementary Data 1). Each of the M1 to M5 domains corresponds to an extended Pfam MSG domain (PF02349) and each domain contains 7 to 8 highly conserved cysteine residues that likely contribute to their secondary structure. The C1 domain corresponds to an extended Pfam Msg2_C domain (PF12373), while the N1, M6 and C2 domains are new Msg signature domains identified after examining Msg protein alignments.

To infer the phylogenetic relationship of *msg* genes, their deduced protein sequences were aligned with MUSCLE[39] and phylogenetic trees were constructed by maximum likelihood (ML) using RAxML (v7.7.8)[40] with model PROTCATWAG and 1,000 bootstrap replicates. Based on domain structure and phylogeny analysis of the *msg* superfamily, we propose a classification of five families, named as Msg-A, Msg-B, Msg-C, Msg-D, and Msg-E (see more details in Fig. 3; Supplementary Table 3 and Supplementary Data 1). The Msg superfamily shows conservation among most Msg families or subfamilies across species, but also species-specific expansions.

Of the five families, the Msg-A family is by far the largest with two of its subfamilies (Msg-A1 representing classical Msg, and Msg-A3, a new subfamily) shared among all three *Pneumocystis* species, while the third subfamily (Msg-A2 or Msr) is absent from *P. jirovecii*. Most members of the Msg-A family contain all nine domains specific for the Msg superfamily (Fig. 3b). The Msg-B family is present only in *P. jirovecii*; most genes encode only 3 Msg signature domains. The Msg-C family is encoded by a tandem array of 6 genes in *P. murina*, with one and two copies in *P. carinii* and *P. jirovecii,* respectively (Supplementary Fig. 4e); each copy encodes 3 Msg signature domains. The Msg-D family is related to the previously reported A12 antigen gene in *P. murina*[41]; this family is encoded by a single gene in *P. murina* and *P. carinii* but expanded to 20 copies in *P. jirovecii,* with the majority encoding six Msg

signature domains. Of note, while the A12 family shares with the kexin family a region of ~50-250 amino acids rich in proline, serine and threonine near the carboxyl end as noted previously[41], no other region is shared between these two families, and none of the 9 Msg signature domains is present in any kexin gene. The Msg-E family is related to two previously reported p55 genes[42,43]; there are 5-7 Msg-E genes in each *Pneumocystis* species, and each of them encodes only one Msg signature domain. Of note, the Msg-A1 family (classic Msg) is the only antigen of *P. jirovecii* that has been well characterized to date, although studies in animal models have suggested that both p55 and A12 genes as well as kexin are antigenic[41,43].

As previously observed in sequencing several telomeric regions in *P. carinii*[36], the *msg* superfamily members in all three *Pneumocystis* species are located almost exclusively in subtelomeric regions. By generating near complete assemblies, we found that two or more members from the same or different families are usually concatenated and are occasionally duplicated with high-level sequence similarity in multiple subtelomeric regions (Supplementary Figs 1 and 2). Such arrangement may facilitate gene recombination as well as transcription (i.e., polycistronic transcription).

Targeted sequencing of *msg*-containing subtelomeric regions in different *P. murina* and *P. carinii* isolates showed no sequence variation between isolates, however different *msg* sequences were found immediately downstream of and in frame with the single copy UCS sequence required for expression[1,44]. These observations support a gene conversion mechanism for *msg* recombination, and no inter-strain variation of the *msg* repertoire in laboratory strains of *P. murina and P. carinii*, as has been suggested from previous studies[37]. In contrast, the *P. jirovecii msg* repertoire shows extensive inter-strain variation[37], and subtelomeres are regions of high diversity between strains (Fig. 1b). RNA-Seq data indicate that all *msg* genes in *P. murina* and *P. carinii* are transcribed in a population of cells (Supplementary Data 1; Supplementary Fig. 14). Of note, the UCS gene in both species is the most highly expressed protein-coding gene, consistent with a very high level of expression of the *msg*-A1 gene subfamily as a whole.

The comprehensive set of Msg superfamily members generated in this study will facilitate characterization of the antigenicity of and immune response to the encoded proteins.

Supplementary Note 5: Metabolic pathway analysis.

Previously, only a limited number of metabolic pathways have been partially characterized in *Pneumocystis,* including those for folate and nucleotide metabolism[45-48], polyamine metabolism[49-51], glucan metabolism[52-55], lipid (mainly sterol) metabolism[56-62], ubiquinone metabolism[63], and chitin metabolism[6,64-66]. Studies on these pathways have focused primarily on identification of the end products and/or only a few key anabolic or catabolic enzymes; none of these pathways has been completely characterized.

The release of the first *P. carinii* genome[7,67] in 2004 and the first *P. jirovecii* genome[8] in 2012 has facilitated the characterization of *Pneumocystis* metabolic pathways. Analysis of both genomes predicted a significant reduction of the amino acid biosynthesis pathways, losses of pathways for the glyoxylate cycle, myo-inositol biosynthesis, thiamine biosynthesis, purine degradation, and nitrogen and sulfur assimilation[5,8,68-70]. These genome data have also facilitated the identification of several genes involved in sterol and beta-glucan metabolism (such as *erg6*, *erg7*, and *eng*). However, the fragmentary and incomplete status of both genomes has prevented a comprehensive and accurate analysis of metabolic pathways, especially depleted pathways.

Since the chromosome-level genome assemblies we generated in this study potentially contain the complete gene set for each species, we mapped all major metabolic pathways (Figs 4 and 5; Supplementary Figs 10 and 11; Supplementary Data 9-19) for all three *Pneumocystis* species using methods essentially as described by Rhind *et al* [11]. Since the *Pneumocystis* genomes are among the smallest in fungi and significant reduction of some metabolic pathways were noted previously[8,68,70,71] and in our initial analysis, we were extra cautious in defining a gene loss. All gene losses in the current report were verified by repeated blast analysis of the annotated protein sets, assembled nucleotide sequences and raw reads we generated, as well as the previously reported sequences for *P. carinii*[7] and *P. jirovecii*[8]. Of note, none of the genes reported as lost in the current study were identified as being present in the previously reported assemblies[7,8].

We found that *Pneumocystis* genomes encode all the enzymes required for GPI anchor synthesis except for one, GPI-protein-acyl-transferase (Cwh43), which is highly conserved within Ascomycota, and in *S. cerevisiae*[72] is responsible for replacing the diacylglycerol moiety of the nascent GPI-anchor moiety with ceramide (Supplementary Data 19). These findings suggest that *Pneumocystis* produces GPI anchored proteins which do not contain ceramide as has been found in some GPI-anchored proteins in *S. cerevisiae*[73].

The gene encoding transaldolase of the pentose phosphate pathway (PPP), which is absent in the previous *P. carinii* genome assembly[69], is also absent in the current *P. carinii* and *P. murina* genomes but present in *P. jirovecii* as well as all other ascomycetes analyzed. PPP is an important source of NADPH for anabolic reactions, and of sugar molecules required for the biosynthesis of nucleic acids and amino acids. While it is possible that the transaldolase gene in rodent *Pneumocystis* could not be identified based on sequence homology due to a low degree of sequence conservation[74], this gene is also missing in *Plasmodium falciparum*[75] and *Cyanidioschyzon merolae*[76]. It is unclear if the transaldolase activities in rodent *Pneumocystis* could be replaced by other enzymes[77].

All three *Pneumocystis* species lack *de novo* synthesis pathways and direct transport systems for phosphatidylinositol, phosphatidylcholine, inositol and choline (Fig. 4; Supplementary Data 12). Nevertheless, these metabolites could be supplied by alternative mechanisms. Each *Pneumocystis* genome encodes the Dnf1-Lem3 flippase complex involved in uptake of external lyso-phosphatidylcholine that can be converted to phosphatidylcholine and subsequently to choline. We also identified a potential transporter (Git1) involved in uptake of external glycerophosphoinositol that could be hydrolyzed into inositol[78], though the enzyme responsible for this hydrolysis has not been definitively identified in *Pneumocystis* or other fungi. Of note, the downstream pathways to make phosphatidylinositol and various inositol phosphate compounds are fully conserved in each *Pneumocystis* species.

A recent report identified inositol transporters in *Pneumocystis*[5], including genes identical to PNEG_01850 and PNEG_00943 in *P. murina*, T552_02044 and T552_00744 in *P. carinii*, and T551_02057 in *P. jirovecii* in this study. Based on our phylogenetic analysis of these genes as well as another closely related but previously unidentified gene (T551_02439 in *P. jirovecii*)[5], these genes are more closely related to glucose transporters than inositol transporters (Supplementary Fig. 9a). It is possible that inositol transporters have been lost in *Pneumocystis.* Inositol is a critical compound needed to synthesize phosphatidylinositol and various inositol phosphates. *Pneumocystis* may rely on an alternative mechanism to obtain inositol as shown in Fig. 4 and discussed above.

As previously reported[68], all *Pneumocystis* species lack key components of the RNA interference (RNAi)-mediated gene silencing system (including the Argonaute and Dicer orthologs); hence, RNA interference–related technologies are unlikely to be of much value in

targeted disruption of genes in *Pneumocystis*. In addition, *Pneumocystis* lacks all three centromere-binding protein CENP-B homologues (Cbp1, Cbh1 and Cbh2), which are present in all sequenced fission yeasts other than *S. japonicus*[11]; these proteins are involved in heterochromatic silencing as well as centromere formation[79,80]. Despite lack of both RNAi- and Cbp1-mediated silencing, *Pneumocystis* shows a relative expansion of the histone deacetylase family (Fig. 2; Supplementary Data 4), suggesting an important role of this gene family in transcription repression in *Pneumocystis*.

Supplementary Note 6: Chitin glycosyl linkage analysis.

After primary digestion with chitinase, terminal N-acetylglucosamine (GlcNAc) and 4-linked GlcNAc were detected in *S. cerevisiae* cell wall samples (as a positive control) in agreement with the glycosyl linkage of chitin reported in *S. cerevisiae*[81], whereas no amino sugar linkage signal was detected in *P. carinii* cell wall samples (Fig. 6b). Pronase and lyticase digestions were performed on *P. carinii* cell walls to digest proteins and glucans, followed by repeat chitinase digestion. Again, no signal corresponding to an amino sugar linkage was detected.

Lyticase digestion homogenized the cell walls of both *P. carinii* and *S. cerevisiae* and each sample gave a strong signal for glucose peaks, indicating the presence of lyticase-sensitive beta-1,3-glucans in each. The glycosyl linkage peaks observed in each sample were terminal-glucose, followed by 3-linked glucose, then 6-linked glucose, with a higher proportion of terminal glucose in *P. carinii* than *S. cerevisiae*. The glucose linkage signals observed for *S. cerevisiae* are in agreement with the known glycosyl linkage of glucan in the *S. cerevisiae* cell wall[82]. The *P. carinii* preparation also contained detectable amounts of 4-linked glucose, which is often a contaminant, though it was seen in both *P. carinii* digests.

Supplementary Note 7: Msg protein glycosylation identification.

Genome analysis suggests that, unlike other fungi, *Pneumocystis* cell wall proteins are not highly mannosylated (Fig. 7; Supplementary Data 21). Since it is not practical to isolate *Pneumocystis* cell wall proteins free of host proteins due to the lack of a reproducible culture method, and Msg is the most abundant surface protein and the only surface protein that has been

well characterized in *Pneumocystis*, we chose to examine glycosylation in *P. carinii* Msg proteins that were affinity purified using an anti-Msg monoclonal antibody.

By liquid chromatography–tandem mass spectrometry (LC-MS/MS) analysis, both the released N-linked profiling and glycopeptide mapping data suggest that M5N2 is a predominant N-glycan component on *P. carinii* Msg (Fig. 7c, d; Supplementary Fig. 13; Supplementary Data 21). The data suggest that M6-M9N2 are also present but the relative abundance of those species are very low compared to M5N2 (Supplementary Fig. 13). Nothing greater than M9N2 was definitively identified. The abundance of those minor species were slightly above-the-noise level in the released N-linked profiling, approaching the limit of detection of mass spectrometry.

Supplementary Methods

Identification of *msg* and kexin genes in subtelomeric regions. In initial genome assemblies for all three *Pneumocystis* species, only partial *msg* genes were present in most scaffold ends, except for the *msg* clusters present in six *P. carinii* scaffolds and one *P. jirovecii* scaffold which were obtained by merging with the previously reported telomere ends[36,83]. Although a large number of raw reads mapped to *msg* genes, they were under-represented in the scaffolds due to high-level sequence identity between different *msg* genes (80-99%), which made accurate assembly difficult.

Identification of msg genes by anchored PCR : Based on known *msg* sequences (*msg*-A1 subfamily, Fig. 3) we designed primers from highly conserved regions, including the putative conserved recombination junction element (CRJE)[1] at the 5' end and a region at the very 3' end. These primers were paired with primers specific for the ends of the targeted scaffolds. All PCR products were subjected to Sanger sequencing; the resulting sequences were added to the targeted scaffolds. This approach extended partial *msg* genes, and identified new *msg* genes at the ends of most scaffolds in *P. murina* and *P. carinii*, including three sets of tandem arrays of 2 or 3 genes (5-10 kb with 95-99% identity), with each set duplicated in 2 or 3 scaffolds in *P. murina* (Supplementary Figs 1a and 2a).

Identification of msg and kexin genes by PacBio sequencing : Following a preliminary study of the ability of PacBio sequencing to provide long reads (>1.5 kb) with high accuracy (~99%) for *msg* genes[84], we utilized PacBio sequencing to identify classical *msg* genes (*msg*-A1 subfamily, Fig. 3) in all three *Pneumocystis* species, *msr* genes (*msg*-A2 subfamily, Fig. 3) in

P. murina and *P. carinii*, and kexin genes in *P. carinii*. For each gene family, two pairs of primers targeting highly conserved regions at the 5' and 3' ends of known *msg* or kexin genes were used in separate PCR reactions to amplify the full- or nearly full-length coding regions (~2-3 kb) from genomic DNA. PCR products were purified and used to construct PacBio DNA circular consensus sequence (CCS) libraries using the PacBio standard 2-3 kb template prep protocol. Each library was sequenced using C2 or C3 chemistry on the PacBio RS or RS II platform[84] at Leidos, Inc. CCS reads were analyzed by a clustering-based computational pipeline as described elsewhere[84]. All contig sequences were corrected using Illumina reads. A proportion (~20-30%) of these contigs could be mapped to the large scaffolds, but the remainder could not, due to the lack of flanking regions, although all could be aligned to Illumina raw reads. The 80 *msg* genes (*msg*-A1 subfamily) for *P. jirovecii* included the 24 *msg* genes previously identified from the same isolate by traditional Sanger sequencing of cloned PCR products[37].

Gene prediction. The *P. murina* and *P. carinii* genomes were annotated using a combination of expression data (RNA-Seq), homology information, and ab initio gene finding methods as previously described[85]. RNA-Seq reads were aligned to the genome using BWA[86] and assembled into transcripts using the inchworm component of Trinity[87]. Inchworm transcripts were aligned to the genome with PASA[88] and used to identify open reading frames (ORFs). The Uniref90 database[89] (downloaded in September 2010 and with fragmentary genes removed) was compared to the genome using BLAST to identify conserved genes. These BLAST alignments were used to predict gene models with Genewise[90]. For ab intio gene-finding, GeneMark-ES[91], which is self-training, was run first. GeneMark models matching full-length PASA RNA-Seq ORFs were used to train Augustus[92], Glimmer[93] and SNAP[94]. The best gene model at each locus was then selected using EVM, which utilizes PASA, Genewise and the ab initio gene predictions as input. PASA-ORFs that did not appear in the EVM gene set were added to produce a draft gene set. PASA was then used to improve gene-model structure, predict splice variants, and add untranslated regions (UTRs) to the draft gene set. The RNA-Seq reads were compared to the draft gene set and assembled with inchworm; these gene-directed assemblies were used by PASA to update structure predictions including UTRs and alternately spliced forms. This second gene-guided assembly step was necessary due to the high gene density in *P.*

murina and *P. carinii*, as genome-guided assemblies included merged transcripts from neighboring genes.

These methods were also used to annotate the RU assembly of *P. jirovecii* from the patient, with the exception that RNA-Seq data was not used. Initial gene sets for the three species were compared to evaluate the consistency of orthologs across the three *Pneumocystis* species, using OrthoMCL[95] to assign orthologs across the three gene sets. Where gene calls appeared to be missing in one or two genomes, we examined all evidence from the individual gene prediction algorithms and RNA-Seq alignments to identify candidate gene models, and where possible added the best model to the gene set. In addition, we examined cases where genes appeared to be missing due to split or merged gene calls; these were corrected where possible. This comparative process resulted in a highly consistent and complete gene set for the three genomes.

Probable mobile elements were removed from the gene set using multiple methods. Predicted genes in conserved repetitive elements were identified using TPSI (http://transposonpsi.sourceforge.net), presence of Pfam domains known to occur in repetitive elements, and a BLAST run against a locally maintained repeat library. Additional repeats were identified using a BLAT alignment of the draft gene set to the genome, requiring 90% nucleotide identity over at least 100 bases; genes matching the genome more than seven times were removed as probable repeats. To ensure this process was not too aggressive, no genes were removed if they included non-repeat Pfam domains.

The final gene set was produced after checking the repeat-filtered updated gene set for in-frame stops, CDS overlaps, multiple Ns in exons, exons ≥ 1500 nt and introns ≤ 20 nt, and corrected manually as needed. The gene sets in *P. carinii* and *P. jirovecii* include all *Pneumocystis*-specific predicted CDSs in previously reported assemblies (Supplementary Table 1).

Comparative and phylogenetic analysis. To identify gene family expansions, protein domain profiles were compiled for all 12 fungal genomes in our comparative set: three *Pneumocystis* species (*P. murina*, *P. carinii* and *P. jirovecii*), *T. deformans*, four *Schizosaccharomycetes* (*S. cryophilus*, *S. japonicus*, *S. octosporus*, and *S. pombe*), *Candida albicans*, *S. cerevisiae*, and two Microsporidia (*E. cuniculi* and *E. intestinalis*) (Supplementary Table 2). The HMMER3

package[96] was used to identify Pfam and TIGRFAM domains using release 27 of Pfam and release 14 of TIGRFAM. KEGG matches (V65) were identified using Blast. Transmembrane regions were predicted using TMHMM (version 2.0)[97], and signal peptide sequences were predicted using SignalP (version 4.0)[2]. These domain profiles were compared between *Pneumocystis* and other fungi to detect significantly enriched or depleted domains using Fisher's exact test with multiple testing correction to compute q-values[98].

To infer the phylogenetic relationship of *Pneumocystis* and related fungi, we identified 413 single copy core orthologs using OrthoMCL[95] in the 12 genomes in our comparative set (Supplementary Table 2). Proteins from each ortholog group were aligned with MUSCLE[39] and the resulting alignments concatenated. A phylogeny was then inferred using RAxML (v7.7.8)[40] with model PROTCATWAG and 1,000 bootstrap replicates.

To identify syntenic regions of conserved gene order between the *Pneumocystis* genomes, we identified all blocks of three or more genes using DAGchainer[99]. Orthologous genes were identified using OrthoMCL and input to DAGchainer to find syntenic regions. In addition to *Pneumocystis*, we also identified syntenic regions with other related fungi in Taphrinomycotina; the block size was set at three to detect such regions. Between *P. murina* and *S. pombe*, we identified 86 syntenic regions encompassing 295 total genes (3.4 genes per block on average). Between *P. murina* and *T. deformans* we detected 36 regions encompassing 121 genes (3.4 genes per block on average); however as the *T. deformans* assembly is highly fragmented, this is likely an under-estimate of syntenic conservation. Syntenic conservation was plotted using functions in R.

The same phylogenetic methods described above were used to analyze genes involved in metabolic pathways. All trees were constructed using protein sequences. Unless otherwise specified in the tree, the protein names from all species shown are used as follows. The proteins from *S. cerevisiae* were indicated by their systematic names alone (seven characters starting with Y, e.g. YFL040W in Supplementary Fig. 9a) or followed with standard abbreviated names (e.g. YGL012W: Erg4 in Supplementary Fig. 11b). For all other species, the proteins were indicated by their locus tags available from NCBI or respective genome databases (Supplementary Table 2). The first two to five characters of the locus tags are species specific, as follows: PNEG for *P. murina*, T552 for *P. carinii*, T551 for *P. jirovecii*, SJAG for *S. japonicus*, SP for *S. pombe*, SPOG for *S. cryophilus*, SOCG for *S. octosporus*, CAL or Ca for *C.*

albicans, TAPDE or Td for *T. deformans,* CIMG for *C. immitis*, Afu or Af for *A. fumigatus*, ECU for *E. cuniculi*, Eint for *E. interstinalis,* Cna for *Cryptococcus neoformans*, and Cnb for *Cryptococcus gattii*.

Supplementary references

1. Keely, S. P., Linke, M. J., Cushion, M. T. & Stringer, J. R. *Pneumocystis murina* MSG gene family and the structure of the locus associated with its transcription. *Fungal Genet Biol* **44**, 905-919, (2007).
2. Petersen, T. N., Brunak, S., von Heijne, G. & Nielsen, H. SignalP 4.0: discriminating signal peptides from transmembrane regions. *Nat Methods* **8**, 785-786, (2011).
3. Kuznets, G. *et al.* A relay network of extracellular heme-binding proteins drives *C. albicans* iron acquisition from hemoglobin. *PLoS Pathog* **10**, e1004407, (2014).
4. Weissman, Z. & Kornitzer, D. A family of *Candida* cell surface haem-binding proteins involved in haemin and haemoglobin-iron utilization. *Mol Microbiol* **53**, 1209-1220, (2004).
5. Porollo, A., Sesterhenn, T. M., Collins, M. S., Welge, J. A. & Cushion, M. T. Comparative genomics of *Pneumocystis* species suggests the absence of genes for myo-inositol synthesis and reliance on inositol transport and metabolism. *MBio* **5**, e01834-01814, (2014).
6. Villegas, L. R., Kottom, T. J. & Limper, A. H. Chitinases in *Pneumocystis carinii* pneumonia. *Med Microbiol Immunol* **201**, 337-348, (2012).
7. Slaven, B. E. *et al.* Draft assembly and annotation of the *Pneumocystis carinii* genome. *J Eukaryot Microbiol* **53 Suppl 1**, S89-91, (2006).
8. Cisse, O. H., Pagni, M. & Hauser, P. M. De novo assembly of the *Pneumocystis jirovecii* genome from a single bronchoalveolar lavage fluid specimen from a patient. *MBio* **4**, e00428-00412, (2012).
9. Cisse, O. H. *et al.* Genome sequencing of the plant pathogen *Taphrina deformans*, the causal agent of peach leaf curl. *MBio* **4**, e00055-00013, (2013).
10. Wood, V. *et al.* The genome sequence of *Schizosaccharomyces pombe*. *Nature* **415**, 871-880, (2002).
11. Rhind, N. *et al.* Comparative functional genomics of the fission yeasts. *Science* **332**, 930-936, (2011).

12. Cherry, J. M. *et al.* *Saccharomyces* Genome Database: the genomics resource of budding yeast. *Nucleic Acids Res* **40**, D700-705, (2012).
13. van het Hoog, M. *et al.* Assembly of the *Candida albicans* genome into sixteen supercontigs aligned on the eight chromosomes. *Genome Biol* **8**, R52, (2007).
14. Katinka, M. D. *et al.* Genome sequence and gene compaction of the eukaryote parasite *Encephalitozoon cuniculi*. *Nature* **414**, 450-453, (2001).
15. Corradi, N., Pombert, J. F., Farinelli, L., Didier, E. S. & Keeling, P. J. The complete sequence of the smallest known nuclear genome from the microsporidian *Encephalitozoon intestinalis*. *Nat Commun* **1**, 77, (2010).
16. Ma, L. *et al.* Sequencing and characterization of the complete mitochondrial genomes of three *Pneumocystis* species provide new insights into divergence between human and rodent *Pneumocystis*. *FASEB J* **27**, 1962-1972, (2013).
17. Cushion, M. T. & Keely, S. P. Assembly and annotation of *Pneumocystis jirovecii* from the human lung microbiome. *MBio* **4**, e00224-00213, (2013).
18. Nahimana, A. *et al.* Determination of the copy number of the nuclear rDNA and beta-tubulin genes of *Pneumocystis carinii f. sp. hominis* using PCR multicompetitors. *J Eukaryot Microbiol* **47**, 368-372, (2000).
19. Tang, X., Bartlett, M. S., Smith, J. W., Lu, J. J. & Lee, C. H. Determination of copy number of rRNA genes in *Pneumocystis carinii f. sp. hominis*. *J Clin Microbiol* **36**, 2491-2494, (1998).
20. Ide, S., Miyazaki, T., Maki, H. & Kobayashi, T. Abundance of ribosomal RNA gene copies maintains genome integrity. *Science* **327**, 693-696, (2010).
21. Torres-Machorro, A. L., Hernandez, R., Cevallos, A. M. & Lopez-Villasenor, I. Ribosomal RNA genes in eukaryotic microorganisms: witnesses of phylogeny? *FEMS Microbiol Rev* **34**, 59-86, (2010).
22. Goffeau, A. *et al.* Life with 6000 genes. *Science* **274**, 546, 563-547, (1996).
23. James, S. A. *et al.* Repetitive sequence variation and dynamics in the ribosomal DNA array of Saccharomyces cerevisiae as revealed by whole-genome resequencing. *Genome research* **19**, 626-635, (2009).
24. Schattner, P., Brooks, A. N. & Lowe, T. M. The tRNAscan-SE, snoscan and snoGPS web servers for the detection of tRNAs and snoRNAs. *Nucleic Acids Res* **33**, W686-689, (2005).

25. Goodenbour, J. M. & Pan, T. Diversity of tRNA genes in eukaryotes. *Nucleic Acids Res* **34**, 6137-6146, (2006).
26. Stevenson, B. S. & Schmidt, T. M. Life history implications of rRNA gene copy number in *Escherichia coli*. *Appl Environ Microbiol* **70**, 6670-6677, (2004).
27. Condon, C., Liveris, D., Squires, C., Schwartz, I. & Squires, C. L. rRNA operon multiplicity in *Escherichia coli* and the physiological implications of rrn inactivation. *J Bacteriol* **177**, 4152-4156, (1995).
28. Kobayashi, T. Regulation of ribosomal RNA gene copy number and its role in modulating genome integrity and evolutionary adaptability in yeast. *Cell Mol Life Sci* **68**, 1395-1403, (2011).
29. Kudla, G., Lipinski, L., Caffin, F., Helwak, A. & Zylicz, M. High guanine and cytosine content increases mRNA levels in mammalian cells. *PLoS Biol* **4**, e180, (2006).
30. Haidaris, C. G., Medzihradsky, O. F., Gigliotti, F. & Simpson-Haidaris, P. J. Molecular characterization of mouse *Pneumocystis carinii* surface glycoprotein A. *DNA Res* **5**, 77-85, (1998).
31. Kutty, G., Ma, L. & Kovacs, J. A. Characterization of the expression site of the major surface glycoprotein of human-derived *Pneumocystis carinii*. *Mol Microbiol* **42**, 183-193, (2001).
32. Wada, M., Sunkin, S. M., Stringer, J. R. & Nakamura, Y. Antigenic variation by positional control of major surface glycoprotein gene expression in *Pneumocystis carinii*. *J Infect Dis* **171**, 1563-1568, (1995).
33. Stringer, J. R. & Keely, S. P. Genetics of surface antigen expression in *Pneumocystis carinii*. *Infect Immun* **69**, 627-639, (2001).
34. Deitsch, K. W., Lukehart, S. A. & Stringer, J. R. Common strategies for antigenic variation by bacterial, fungal and protozoan pathogens. *Nat Rev Microbiol* **7**, 493-503, (2009).
35. Gigliotti, F. & Haidaris, C. G. Antigenic characterization of *Pneumocystis carinii*. *Semin Respir Infect* **13**, 313-322, (1998).
36. Keely, S. P. *et al.* Gene arrays at *Pneumocystis carinii* telomeres. *Genetics* **170**, 1589-1600, (2005).
37. Kutty, G., Maldarelli, F., Achaz, G. & Kovacs, J. A. Variation in the major surface glycoprotein genes in *Pneumocystis jirovecii*. *J Infect Dis* **198**, 741-749, (2008).

38. Huang, S. N., Angus, C. W., Turner, R. E., Sorial, V. & Kovacs, J. A. Identification and characterization of novel variant major surface glycoprotein gene families in rat *Pneumocystis carinii*. *J Infect Dis* **179**, 192-200, (1999).
39. Edgar, R. C. MUSCLE: multiple sequence alignment with high accuracy and high throughput. *Nucleic Acids Res* **32**, 1792-1797, (2004).
40. Stamatakis, A. RAxML-VI-HPC: maximum likelihood-based phylogenetic analyses with thousands of taxa and mixed models. *Bioinformatics* **22**, 2688-2690, (2006).
41. Wells, J., Gigliotti, F., Simpson-Haidaris, P. J. & Haidaris, C. G. Epitope mapping of a protective monoclonal antibody against *Pneumocystis carinii* with shared reactivity to *Streptococcus pneumoniae* surface antigen PspA. *Infect Immun* **72**, 1548-1556, (2004).
42. Ma, L., Kutty, G., Jia, Q. & Kovacs, J. A. Characterization of variants of the gene encoding the p55 antigen in *Pneumocystis* from rats and mice. *J Med Microbiol* **52**, 955-960, (2003).
43. Smulian, A. G., Stringer, J. R., Linke, M. J. & Walzer, P. D. Isolation and characterization of a recombinant antigen of *Pneumocystis carinii*. *Infect Immun* **60**, 907-915, (1992).
44. Keely, S. P. & Stringer, J. R. Complexity of the MSG gene family of *Pneumocystis carinii*. *BMC Genomics* **10**, 367, (2009).
45. Edman, U., Edman, J. C., Lundgren, B. & Santi, D. V. Isolation and expression of the *Pneumocystis carinii* thymidylate synthase gene. *Proc Natl Acad Sci U S A* **86**, 6503-6507, (1989).
46. Kovacs, J. A. *et al.* Characterization of de novo folate synthesis in *Pneumocystis carinii* and *Toxoplasma gondii*: potential for screening therapeutic agents. *J Infect Dis* **160**, 312-320, (1989).
47. Vestereng, V. H. & Kovacs, J. A. Inability of *Pneumocystis* organisms to incorporate bromodeoxyuridine suggests the absence of a salvage pathway for thymidine. *Microbiology* **150**, 1179-1182, (2004).
48. Ye, D., Lee, C. H. & Queener, S. F. Differential splicing of *Pneumocystis carinii f. sp. carinii* inosine 5'-monophosphate dehydrogenase pre-mRNA. *Gene* **263**, 151-158, (2001).
49. Lasbury, M. E. *et al.* Polyamine-mediated apoptosis of alveolar macrophages during *Pneumocystis* pneumonia. *J Biol Chem* **282**, 11009-11020, (2007).

50. Liao, C. P. *et al. Pneumocystis* mediates overexpression of antizyme inhibitor resulting in increased polyamine levels and apoptosis in alveolar macrophages. *J Biol Chem* **284**, 8174-8184, (2009).
51. Lipschik, G. Y., Masur, H. & Kovacs, J. A. Polyamine metabolism in *Pneumocystis carinii*. *J Infect Dis* **163**, 1121-1127, (1991).
52. Kottom, T. J., Hebrink, D. M., Jenson, P. E., Gudmundsson, G. & Limper, A. H. Evidence for pro-inflammatory beta-1,6 glucans in the *Pneumocystis* cell wall. *Infect Immun* **83**, 2816-2826, (2015).
53. Kottom, T. J. & Limper, A. H. Cell wall assembly by *Pneumocystis carinii.* Evidence for a unique gsc-1 subunit mediating beta -1,3-glucan deposition. *J Biol Chem* **275**, 40628-40634, (2000).
54. Kutty, G., Davis, A. S., Ma, L., Taubenberger, J. K. & Kovacs, J. A. *Pneumocystis* encodes a functional endo-beta-1,3-glucanase that is expressed exclusively in cysts. *J Infect Dis* **211**, 719-728, (2015).
55. Villegas, L. R., Kottom, T. J. & Limper, A. H. Characterization of PCEng2, a {beta}-1,3-endoglucanase homolog in *Pneumocystis carinii* with activity in cell wall regulation. *Am J Respir Cell Mol Biol* **43**, 192-200, (2010).
56. Florin-Christensen, M. *et al.* Occurrence of specific sterols in *Pneumocystis carinii*. *Biochem Biophys Res Commun* **198**, 236-242, (1994).
57. Joffrion, T. M. & Cushion, M. T. Sterol biosynthesis and sterol uptake in the fungal pathogen *Pneumocystis carinii*. *FEMS Microbiol Lett* **311**, 1-9, (2010).
58. Kaneshiro, E. S. *et al.* Pneumocysterol [(24Z)-ethylidenelanost-8-en-3beta-ol], a rare sterol detected in the opportunistic pathogen *Pneumocystis carinii* hominis: structural identity and chemical synthesis. *Proc Natl Acad Sci U S A* **96**, 97-102, (1999).
59. Kaneshiro, E. S., Johnston, L. Q., Nkinin, S. W., Romero, B. I. & Giner, J. L. Sterols of *Saccharomyces cerevisiae* erg6 Knockout Mutant Expressing the *Pneumocystis carinii* S-Adenosylmethionine:Sterol C-24 Methyltransferase. *J Eukaryot Microbiol* **62**, 298-306, (2015).
60. Kaneshiro, E. S. *et al. Pneumocystis carinii* erg6 gene: sequencing and expression of recombinant SAM:sterol methyltransferase in heterologous systems. *J Eukaryot Microbiol* **Suppl**, 144S-146S, (2001).

61. Nkinin, S. W. *et al.* *Pneumocystis carinii* sterol 14alpha-demethylase activity in *Saccharomyces cerevisiae* erg11 knockout mutant: sterol biochemistry. *J Eukaryot Microbiol* **58**, 383-392, (2011).
62. Yoshikawa, H., Morioka, H. & Yoshida, Y. Freeze-fracture localization of filipin-sterol complexes in plasma- and cyto-membranes of *Pneumocystis carinii*. *J Protozool* **34**, 131-137, (1987).
63. Basselin, M., Hunt, S. M., Abdala-Valencia, H. & Kaneshiro, E. S. Ubiquinone synthesis in mitochondrial and microsomal subcellular fractions of *Pneumocystis* spp.: differential sensitivities to atovaquone. *Eukaryot Cell* **4**, 1483-1492, (2005).
64. Rapaka, R. R. *et al.* Conserved natural IgM antibodies mediate innate and adaptive immunity against the opportunistic fungus *Pneumocystis murina*. *J Exp Med* **207**, 2907-2919, (2010).
65. Roth, A., Wecke, J., Karsten, V. & Janitschke, K. Light and electron microscopy study of carbohydrate antigens found in the electron-lucent layer of *Pneumocystis carinii* cysts. *Parasitol Res* **83**, 177-184, (1997).
66. Walker, A. N., Garner, R. E. & Horst, M. N. Immunocytochemical detection of chitin in *Pneumocystis carinii*. *Infect Immun* **58**, 412-415, (1990).
67. Cushion, M. T. Comparative genomics of *Pneumocystis carinii* with other protists: implications for life style. *J Eukaryot Microbiol* **51**, 30-37, (2004).
68. Cisse, O. H., Pagni, M. & Hauser, P. M. Comparative genomics suggests that the human pathogenic fungus *Pneumocystis jirovecii* acquired obligate biotrophy through gene loss. *Genome Biol Evol* **6**, 1938-1948, (2014).
69. Cushion, M. T. *et al.* Transcriptome of *Pneumocystis carinii* during fulminate infection: carbohydrate metabolism and the concept of a compatible parasite. *PLoS One* **2**, e423, (2007).
70. Hauser, P. M. *et al.* Comparative genomics suggests that the fungal pathogen *Pneumocystis* is an obligate parasite scavenging amino acids from its host's lungs. *PLoS One* **5**, e15152, (2010).
71. Hauser, P. M. Genomic insights into the fungal pathogens of the genus *Pneumocystis*: obligate biotrophs of humans and other mammals. *PLoS Pathog* **10**, e1004425, (2014).

72. Umemura, M., Fujita, M., Yoko, O. T., Fukamizu, A. & Jigami, Y. *Saccharomyces cerevisiae* CWH43 is involved in the remodeling of the lipid moiety of GPI anchors to ceramides. *Mol Biol Cell* **18**, 4304-4316, (2007).
73. Fankhauser, C. *et al.* Structures of glycosylphosphatidylinositol membrane anchors from *Saccharomyces cerevisiae*. *J Biol Chem* **268**, 26365-26374, (1993).
74. Samland, A. K. *et al.* Conservation of structure and mechanism within the transaldolase enzyme family. *FEBS J* **279**, 766-778, (2012).
75. Bozdech, Z. & Ginsburg, H. Data mining of the transcriptome of *Plasmodium falciparum*: the pentose phosphate pathway and ancillary processes. *Malar J* **4**, 17, (2005).
76. Moriyama, T., Sakurai, K., Sekine, K. & Sato, N. Subcellular distribution of central carbohydrate metabolism pathways in the red alga *Cyanidioschyzon merolae*. *Planta* **240**, 585-598, (2014).
77. Nakahigashi, K. *et al.* Systematic phenome analysis of *Escherichia coli* multiple-knockout mutants reveals hidden reactions in central carbon metabolism. *Mol Syst Biol* **5**, 306, (2009).
78. Patton-Vogt, J. Transport and metabolism of glycerophosphodiesters produced through phospholipid deacylation. *Biochim Biophys Acta* **1771**, 337-342, (2007).
79. Cam, H. P., Noma, K., Ebina, H., Levin, H. L. & Grewal, S. I. Host genome surveillance for retrotransposons by transposon-derived proteins. *Nature* **451**, 431-436, (2008).
80. Nakagawa, H. *et al.* Fission yeast CENP-B homologs nucleate centromeric heterochromatin by promoting heterochromatin-specific histone tail modifications. *Genes Dev* **16**, 1766-1778, (2002).
81. Kollar, R., Petrakova, E., Ashwell, G., Robbins, P. W. & Cabib, E. Architecture of the yeast cell wall. The linkage between chitin and beta(1-->3)-glucan. *J Biol Chem* **270**, 1170-1178, (1995).
82. Lipke, P. N. & Ovalle, R. Cell wall architecture in yeast: new structure and new challenges. *J Bacteriol* **180**, 3735-3740, (1998).
83. Mei, Q. *et al.* Characterization of major surface glycoprotein genes of human *Pneumocystis carinii* and high-level expression of a conserved region. *Infect Immun* **66**, 4268-4273, (1998).

84. Jiao, X. *et al.* A Benchmark Study on Error Assessment and Quality Control of CCS Reads Derived from the PacBio RS. *J Data Mining Genomics Proteomics* **4**, (2013).
85. Haas, B. J., Zeng, Q., Pearson, M. D., Cuomo, C. A. & Wortman, J. R. Approaches to Fungal Genome Annotation. *Mycology* **2**, 118-141, (2011).
86. Li, H. & Durbin, R. Fast and accurate long-read alignment with Burrows-Wheeler transform. *Bioinformatics* **26**, 589-595, (2010).
87. Grabherr, M. G. *et al.* Full-length transcriptome assembly from RNA-Seq data without a reference genome. *Nat Biotechnol* **29**, 644-652, (2011).
88. Haas, B. J. *et al.* Automated eukaryotic gene structure annotation using EVidenceModeler and the Program to Assemble Spliced Alignments. *Genome Biol* **9**, R7, (2008).
89. Suzek, B. E., Huang, H., McGarvey, P., Mazumder, R. & Wu, C. H. UniRef: comprehensive and non-redundant UniProt reference clusters. *Bioinformatics* **23**, 1282-1288, (2007).
90. Birney, E., Clamp, M. & Durbin, R. GeneWise and Genomewise. *Genome research* **14**, 988-995, (2004).
91. Ter-Hovhannisyan, V., Lomsadze, A., Chernoff, Y. O. & Borodovsky, M. Gene prediction in novel fungal genomes using an ab initio algorithm with unsupervised training. *Genome research* **18**, 1979-1990, (2008).
92. Stanke, M. & Waack, S. Gene prediction with a hidden Markov model and a new intron submodel. *Bioinformatics* **19 Suppl 2**, ii215-225, (2003).
93. Majoros, W. H., Pertea, M. & Salzberg, S. L. TigrScan and GlimmerHMM: two open source ab initio eukaryotic gene-finders. *Bioinformatics* **20**, 2878-2879, (2004).
94. Korf, I. Gene finding in novel genomes. *BMC Bioinformatics* **5**, 59, (2004).
95. Li, L., Stoeckert, C. J., Jr. & Roos, D. S. OrthoMCL: identification of ortholog groups for eukaryotic genomes. *Genome research* **13**, 2178-2189, (2003).
96. Eddy, S. R. Accelerated Profile HMM Searches. *PLoS Comput Biol* **7**, e1002195, (2011).
97. Krogh, A., Larsson, B., von Heijne, G. & Sonnhammer, E. L. Predicting transmembrane protein topology with a hidden Markov model: application to complete genomes. *J Mol Biol* **305**, 567-580, (2001).
98. Storey, J. D. & Tibshirani, R. Statistical significance for genomewide studies. *Proc Natl Acad Sci U S A* **100**, 9440-9445, (2003).

99. Haas, B. J., Delcher, A. L., Wortman, J. R. & Salzberg, S. L. DAGchainer: a tool for mining segmental genome duplications and synteny. *Bioinformatics* **20**, 3643-3646, (2004).
